# Bayesian Calibration of Electrophysiology Models Using Restitution Curve Emulators

**DOI:** 10.3389/fphys.2021.693015

**Published:** 2021-07-22

**Authors:** Sam Coveney, Cesare Corrado, Jeremy E. Oakley, Richard D. Wilkinson, Steven A. Niederer, Richard H. Clayton

**Affiliations:** ^1^Insigneo Institute for In-Silico Medicine and Department of Computer Science, University of Sheffield, Sheffield, United Kingdom; ^2^Division of Imaging Sciences and Biomedical Engineering, King's College London, London, United Kingdom; ^3^School of Mathematics and Statistics, University of Sheffield, Sheffield, United Kingdom; ^4^School of Mathematical Sciences, University of Nottingham, Nottingham, United Kingdom

**Keywords:** restitution, electrophysiology, cardiology, Gaussian processes, emulation, sensitivity analysis, calibration, Bayesian

## Abstract

Calibration of cardiac electrophysiology models is a fundamental aspect of model personalization for predicting the outcomes of cardiac therapies, simulation testing of device performance for a range of phenotypes, and for fundamental research into cardiac function. Restitution curves provide information on tissue function and can be measured using clinically feasible measurement protocols. We introduce novel “restitution curve emulators” as probabilistic models for performing model exploration, sensitivity analysis, and Bayesian calibration to noisy data. These emulators are built by decomposing restitution curves using principal component analysis and modeling the resulting coordinates with respect to model parameters using Gaussian processes. Restitution curve emulators can be used to study parameter identifiability via sensitivity analysis of restitution curve components and rapid inference of the posterior distribution of model parameters given noisy measurements. Posterior uncertainty about parameters is critical for making predictions from calibrated models, since many parameter settings can be consistent with measured data and yet produce very different model behaviors under conditions not effectively probed by the measurement protocols. Restitution curve emulators are therefore promising probabilistic tools for calibrating electrophysiology models.

## 1. Introduction

Cardiac electrophysiology models reconstruct electrical activation of the heart at cell, tissue, and organ scale. Biophysically detailed cardiac cell models aim to represent how ion channels, pumps, and exchangers in the cell membrane co-operate to produce an action potential and calcium transient (Fink et al., 2011). While they can be a good mechanistic representation, these models have large numbers of parameters, which may not all be identifiable from data (Whittaker et al., 2020), and when combined with a tissue model there are complex relationships between model parameter sets and emergent properties such as restitution or spiral wave stability (Cherry and Evans, 2008). Alongside biophysically detailed models of cardiac cellular electrophysiology, phenomenological models have been developed that capture action potential shape and rate dependence without an explicit representation of ion channel behavior (Fenton and Karma, 1998; Mitchell and Schaeffer, 2003; Corrado and Niederer, 2016). These models have fewer parameters than more detailed models and can be solved relatively quickly, but the association between model parameters and emergent properties remains complex (Fenton et al., 2002).

Cardiac models have the potential to be used to guide interventions in the clinic (Niederer et al., 2019). Applications in the clinical setting will require models that are not only fast running, but can also be calibrated quickly from clinical measurements to create personalized models (Sermesant et al., 2012; Boyle et al., 2021). The phenomenological Mitchell-Schaeffer model (Mitchell and Schaeffer, 2003), with relatively few parameters, may be a good candidate in this regard (Relan et al., 2010, 2011; Corrado et al., 2017). Clinical data are typically noisy and sparse so recent developments have included a set of approaches that take into account uncertainties in the data to create probabilistic models (Konukoglu et al., 2011; Coveney et al., 2020; Dhamala et al., 2020), as well as new models designed with uncertainty in mind (Pathmanathan et al., 2019).

Parameter inference methods for cardiac cell models include gradient descent (Dokos and Lovell, 2004), genetic algorithms (Groenendaal et al., 2015; Krogh-Madsen et al., 2016; Cairns et al., 2017; Smirnov et al., 2020), particle swarm (Loewe et al., 2015), multivariate regression (Sarkar and Sobie, 2010), and Markov chain Monte Carlo (Johnstone et al., 2016). In “population of models” approaches, parameter sets that are consistent with data are retained from an initially larger design spanning the parameter space (Muszkiewicz et al., 2015). However, these methods do not obtain a posterior probability distribution for the model parameters, although there have been some efforts to overcome this limitation (Tixier et al., 2017; Lawson et al., 2018). Likewise, history matching approaches accounting for uncertainty still only find plausible parameterizations of cardiac models given data (Coveney and Clayton, 2018).

Inference of model parameters from clinical data is challenging because it is difficult to measure action potentials directly in the clinical setting, especially in atrial tissue. In the clinical setting, the rate dependence of local activation time (LAT) and effective refractory period (ERP) can be measured directly at different locations with pacing at different intervals. LAT can be used to infer conduction velocity (CV) restitution, and ERP restitution is related to action potential duration (APD) restitution. While calibration can aim to find a single “best fit” to the data (Corrado et al., 2017), in general there are many parameter configurations that are consistent with observed data. Two important questions therefore arise: are parameters identifiable from restitution curve data, and can a posterior distribution on model parameters can be obtained from this data?

*Markov chain Monte Carlo* (MCMC) can be used to obtain samples from the posterior distribution, but requires large numbers of simulated restitution curves to be obtained. APD, CV, and ERP restitution curves can be time consuming to compute because they require many solves of a tissue model at different diastolic intervals. Furthermore, these large numbers of simulations cannot be pre-calculated since they must be drawn with posterior probability determined by the data. Expensive simulations can be supplemented with fast-running emulators, sometimes called surrogate models, which can be used to map model inputs onto outputs. Gaussian process (GP) emulators, which provide a prediction and corresponding prediction uncertainty, can be effective emulators of complex computer models (Conti and O'Hagan, 2010). GP emulators have been used for sensitivity analysis (Chang et al., 2015; Coveney and Clayton, 2020) and history matching (Coveney and Clayton, 2018) of cardiac cell models, and for models of cardiac tissue (Dhamala et al., 2020; Lawson et al., 2020) and mechanics (Longobardi et al., 2020). Emulators are conditioned on pre-calculated simulator data, but since they can make predictions at new inputs they are ideal tools for MCMC.

In this paper we describe how to build *Restitution Curve Emulators* (RCEs) for APD, CV, and ERP restitution curves. We chose to base this study on the phenomenological modified Mitchell-Schaeffer (mMS) model (Corrado and Niederer, 2016), since this can be considered a minimal model for capturing the shape and restitution of the cardiac action potential. The emulation of restitution curves using Gaussian processes requires a dimensionality reduction stage using principal component analysis, allowing the curves to be modeled with a small number of independent Gaussian processes. Furthermore, we develop a novel likelihood function for ERP observations. RCEs can then be used with MCMC to obtain the posterior distribution of model parameters given noisy data. The structure of the paper is as follows. First we briefly describe the mMS cellular electrophysiology model, and how it was implemented in a tissue strip model to calculate restitution curves. Next we explain how these restitution curves were decomposed, and how emulators (RCEs) of these curves were constructed. We conduct sensitivity analysis using emulation, showing the effects of the parameters on the principal modes of variation of the curves. Finally we show how these emulators can be used to obtain the posterior distribution of model parameters given noisy measurements of CV, APD, and ERP restitutions.

## 2. Methods

In sections 2.1, 2.2, and 2.3, we explain how restitution curves were simulated, how dimensionality reduction was performed, and how Restitution Curve Emulators were built. In section 2.4, we explain how RCEs can be used for Sensitivity Analysis (SA). In section 2.5, we show how RCEs can be used for probabilistic calibration using uncertain measurements of APD, CV, and ERP restitution curves.

### 2.1. Electrophysiology Model

The mMS cell model (Corrado and Niederer, 2016) was incorporated into a monodomain model of tissue electrophysiology with isotropic diffusion, expressed in the following equations:


(1) $$\begin{array}{l} \frac{\partial V_{m}}{\partial t}=D\nabla^{2}V_{m}+h\frac{V_{m}\left(V_{m}-V_{gate}\right)\left(1-V_{m}\right)}{\tau_{in}}\\ \;-\left(1-h\right)\frac{V_{m}}{\tau_{out}}+J_{stim} \end{array}$$

(2) $$\begin{array}{l} \frac{\partial h}{\partial t}=\{\begin{array}{ll} \left(1-h\right)/\tau_{open} & \text{if }V_{m}\leq V_{gate}\\ -h/\tau_{close} & \text{otherwise} \end{array} \end{array}$$

where the two states are *V*_*m*_, a normalized membrane voltage varying between 0 and 1 (note *V*_*m*_ = *V*_*m*_(**z**, *t*) where **z** indicates space), and *h*, a gating parameter that controls recovery of excitability. We fixed the excitation threshold *V*_*gate*_ to 0.1, leaving five remaining parameters: the tissue diffusion coefficient *D*, and time constants τ_*in*_, τ_*close*_, τ_*out*_, τ_*open*_, which correspond to the initiation, plateau, decay, and recovery phases of the cardiac action potential (Mitchell and Schaeffer, 2003). We reparameterized the model by substituting *D* and τ_*close*_ with the transformed parameters *CV*_*max*_ and *APD*_*max*_, based on asymptotic expressions of model behavior:


(3) $$\begin{array}{l} CV_{max}=0.5\left(1-2V_{gate}\right)\sqrt{2D/\tau_{in}} \end{array}$$

(4) $$\begin{array}{l} APD_{max}=\tau_{close}\log\left(1+\tau_{out}{\left(1-V_{gate}\right)}^{2}/4\tau_{in}\right) \end{array}$$

This reparameterization means that propagating action potentials can be generated for values of transformed parameters within a 5d hypercube, whereas the region of the original parameter space from which propagating action potentials could be generated was relatively small and highly concave. We refer to the transformed parameters {*CV*_*max*_, τ_*in*_, τ_*out*_, τ_*open*_, *APD*_*max*_} as parameters from now on.

We used openCARP (Plank et al., 2021) to solve these equations and obtain CV, APD, and ERP restitution curves for different sets of transformed parameters in a thin strip of simulated tissue. These simulations used a 24 × 0.6 mm triangular finite element mesh, with triangle edges of 0.3 mm and no-flux boundary conditions, and were solved using a time step of 0.1 ms, with a factor 10 smaller time-step for the mMS model. Simulation geometries such as “cables” of 3D elements can be used to reduce simulation time. However, simulation behavior does depend on element type and space-time discretization, so ideally calibration of computational models should utilize restitution curves generated with a comparable simulation setup. Our choices here were motivated by settings that we typically utilize for atrial simulations with the mMS model.

**Figure 1:**
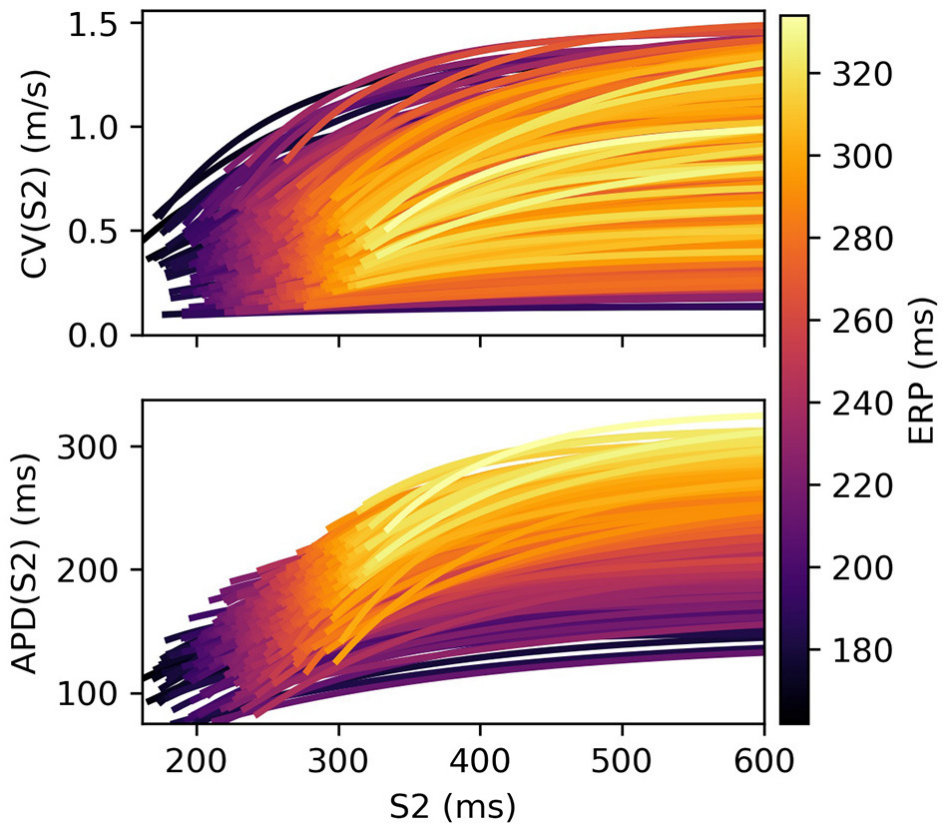
S2 restitution curves for S1: 600 ms for CV(S2) and APD(S2), colored by ERP(S1: 600), and plotted only for S2 > ERP(S1: 600) for clarity.

Restitution curves for S1S2 pacing, representing the variation of either CV or APD with respect to S1S2 intervals for a given S1 interval, which we denote by CV(S2) and APD(S2), respectively (thus abbreviating “S1S2 interval” with S2), were obtained by pacing from one end of the tissue strip (along the shorter edge) using an S1S2 pacing protocol. Example restitution curves are shown in Figure 1 below. CV was determined in the central region of the strip from activation times obtained using a relative threshold of 0.7, and APD was determined as the duration between this latter threshold and a relative threshold of 0.1 (i.e., *APD* ≡ *APD*_90_, the time required for 90% repolarization). ERP was determined as the largest S1S2 interval for which the S2 stimulus did not result in propagation reaching the strip center. For a given set of parameters (homogeneous across the strip) and S1 interval, the strip model was run for integer values of S1S2 interval (in ms), chosen dynamically in order to bisect ERP to a 1 ms resolution. The strip was paced with eight S1 beats and the model state shortly after the final S1 beat was saved (we found no appreciable difference using 16 beats). The S1S2 interval was then varied using Algorithm 1 (reloading the saved model state) until ERP was determined. We set the initial bracketing values for ERP to be 100 and 2,000 ms, which helped ensure that data was collected in both the asymptotic limit of high S1S2 interval, while focusing most observations at S1S2 intervals nearer to ERP. We also consider ERP(S1) restitution curves in this paper, which are curves of ERP for different S1 interval.

### 2.2. Dimensionality Reduction

To build Restitution Curve Emulators requires that we obtain simulation results (outputs) for a space-filling design of parameters (inputs). We generated a Latin hypercube design of 500 “points” in parameter space, optimized with respect to a maximin criterion across 10^4^ designs, in the ranges *CV*_*max*_ 0.1–1.5 m/s, τ_*in*_ 0.01–0.30 ms, τ_*out*_ 1–30 ms, τ_*open*_ 65–215 ms, *APD*_*max*_ 120–270 ms, which were chosen so that the range of corresponding tissue behaviors include, and go sightly beyond, physiologically plausible values (this helps ensure that the output space of plausible values is well sampled). The simulation described above was run for each parameter vector for a specific S1 interval.

**Algorithm 1:**
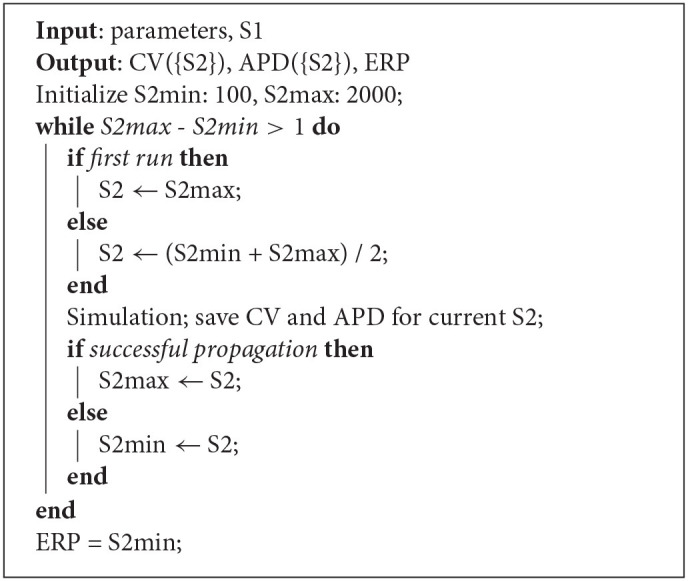
Strip simulation algorithm for S1S2 pacing. For a given S1 interval and set of model parameters, the simulation determines CV(S2) and APD(S2) restitution curves and ERP.

The S2 restitution curves (outputs) obtained from the simulations are obtained for a subset of S1S2 intervals due to the bisection method. Furthermore, since measurements at S1S2 intervals below ERP cannot be made, the restitution curves would not all share the same set of S1S2 interval even if the algorithm was run for a predetermined set of S1S2 intervals. We can fit the restitution curve data to an analytic expression for restitution, which allows us to resample the restitution curves to a common S1S2 interval resolution. For the mMS model, we fitted the following expression to the data using non-linear fitting methods (scipy.optimize.curve_fit function in this case):


(5) $$\begin{array}{l} F\left(S2\right)=a\left(1-b\exp\left(-S2/c\right)\right), \end{array}$$

which fits the data with negligible residuals. The advantage of fitting an analytic expression to each curve is that curves can be extrapolated to obtain “virtual” values for S1S2 interval < ERP, required for PCA since all curves must have the same dimensionality. We refer to this region of restitution curves as “virtual” in analogy with a virtual image in optics, found by tracing real rays from a mirror backwards to a perceived origin behind the mirror from which light rays cannot actually emerge. We chose S1S2 intervals from 160 to 600 ms at 1 ms resolution (corresponding to the highest clinical pacing resolution). For convenience, the “fitting” and “prediction” stages of this resampling are split, such that the simulator fits and returns these coefficients, while prediction happens “outside” of the simulator. (This division is simply for convenience, since the simulator is then a black box that always returns the same number of outputs, rather than variable length arrays depending on the path taken by the bisection algorithm).

We emphasize here that the *only* purpose of Equation (5) is to calculate S2 restitution curves at a common resolution, after which it is never used again. We discuss why emulation of Diastolic Interval (DI) curves, (where *DI* = *S*2 − *ERP* such that the curves would have no virtual region) is *not* a good choice for calibration in Section 3.5. Equation (5) is a non-linear compression of the data into three dimensions, but we found that attempts to predict the coefficients *a, b, c* from the model parameters (followed by application of Equation 5) gave inferior results to the emulation method we present in this paper. Importantly, for *any* re-parameterization of Equation (5), the intrinsic non-linearity means that coefficient emulation with a Gaussian process emulator results in restitution curve emulators that are not Gaussian processes; this significantly complicates exploratory analysis, sensitivity analysis, and calibration, since posterior sampling would be required in all cases to make any predictions. Furthermore, characterizing these predictions would be more difficult, since the mean, median, and mode of these predictions would all be different, and the distribution spread would not be summarized by only the second-order moment, i.e., variance.

We discuss dimensionality reduction here in terms of S2 restitution curves (where for convenience of notation S2 ≡ S1S2 interval). The resulting set of resampled restitution curves can be thought of as a stack of 1D images (1 image per parameter choice) with 1ms wide pixels centered on S1S2 interval, where the pixel intensity represents either CV(S2) or APD(S2). This analogy makes it clear that although each curve has 440 dimensions, the intensity values in many neighboring pixels are highly correlated. Principal Component Analysis (PCA) can be used to find an ordered set of orthogonal directions/axes in this high dimensional space along which the variance between different images is largest. We perform PCA via Singular Value Decomposition (SVD) using sklearn.decomposition.PCA, first subtracting the mean and without scaling the data since the units are identical across dimensions (m/s for CV, ms for APD, ms for ERP) and amplitude of variation is intrinsically important. We obtain a set of right singular vectors (equivalent to eigenvectors) Φ_*c*_(*S*2) for *c* = 1…*C*, where 1 ≤ *C* ≤ *n* for a dataset of size *n* (usually *C*≪*n*). Each restitution curve can be projected onto these axis to obtain the coordinate of that curve in this new space. Each curve can then be expressed with a linear combination of the eigenvectors Φ_*c*_ plus the mean Φ_0_:


(6) $$\begin{array}{l} F\left(S2\right)\approx \Phi_{0}\left(S2\right)+\underset{c}{\sum }f_{c}\cdot \Phi_{c}\left(S2\right), \end{array}$$

where the sum is truncated to keep only the “principal components” accounting for the majority of the variation across the dataset (determined from the corresponding eigenvalues).

For ERP(S1) curves, obtained by running the simulator for a range of S1 intervals, we perform PCA on the data without any resampling in S1. It is interesting to consider that fitting a functional form to ERP(S1) data would allow extrapolation of ERP curves into a virtual region [e.g., if ERP(S1:375) = 360 then ERP(S1:350) is not defined, since the tissue cannot support this S1 pacing, but a virtual value could be defined from a functional fit to the valid ERP(S1) values]. This would allow for keeping additional simulation runs in the emulation dataset that would otherwise be discarded because the ERP(S1) vector would be undefined for some S1, preventing inclusion of those results in PCA for ERP(S1). We do not consider this matter further here, instead opting to discard certain simulation runs from our emulation dataset if some ERP(S1) could not be defined [this means that the ERP(S1) dataset will include only data, i.e., parameters and corresponding ERP(S1) values, for which ERP(S1) can be defined for all S1 values in our dataset].

### 2.3. Restitution Curve Emulators

To create surrogate models that predict the restitution curves *F*(*S*2) from the model parameters **x**, we model each coordinate in Equation (6) as *f*_*c*_ ≡ *f*_*c*_(**x**) using a Gaussian process (Higdon et al., 2008; Wilkinson, 2010), with explicit basis functions modeling the GP mean (Conti and O'Hagan, 2010). We drop the index *c* to reduce clutter in the following equations, as the same type of model is built for all coordinates. For increased numerical stability and model regularization, we assume that the coordinates obtained from PCA are potentially noisy, therefore we denote these values (for a particular *c*) by *y* and the model for these coordinates by *f*. For *n* training data {**x_i_**, *y*_*i*_}, where *i* = 1…*n*, we then have:


(7) $$\begin{array}{l} y\sim N\left(f,{\left(\nu \sigma \right)}^{2}\right) \end{array}$$

(8) $$\begin{array}{l} f|\beta ,\sigma ,\theta \sim N\left(H^{T}\beta ,\sigma^{2}A\right) \end{array}$$

(9) $$\begin{array}{l} H=\left(h(x_{1}),\ldots ,h(x_{n})\right) \end{array}$$

(10) $$\begin{array}{l} A_{ij}=k\left(x_{i},x_{j},\theta \right) \end{array}$$

where the mean function depends on basis functions **h(·)** and basis coefficients ***β***, and the kernel function *k*(·, ·, θ) depends on hyperparameters ***θ*** (we have factored out the amplitude σ^2^). Note that the covariance matrix of the training data **y** is then given by $\sigma^{2}A_{y}=\sigma^{2}\left(A+\nu^{2}I_{n}\right)$, such that the (unscaled) covariance matrix elements *A*_*ij*_ depend on **x_i_** and **x_j_**.

We optimize the hyperparameters ***θ*** and ν (distinct from the model parameters **x**) by maximizing the (marginal) log likelihood L. Denoting *n* and *q* as the number of data points and basis functions, respectively, the basis coefficients and covariance amplitude are integrated out to give $\hat{\beta }$ and ${\hat{\sigma }}^{2}$, respectively (Oakley, 1999; Rasmussen and Williams, 2006; Conti and O'Hagan, 2010), giving:


(11) $$\begin{array}{l} \hat{\beta }={\left(HA_{y}^{-1}H^{T}\right)}^{-1}HA_{y}^{-1}\text{y} \end{array}$$

(12) $$\begin{array}{l} {\hat{\sigma }}^{2}={\left(n-q\right)}^{-1}{\left(\text{y}-H^{T}\hat{\beta }\right)}^{T}A_{y}^{-1}\left(\text{y}-H^{T}\hat{\beta }\right) \end{array}$$

(13) $$\begin{array}{l} L=-\frac{1}{2}\left(\log|A_{y}|+\log|HA_{y}^{-1}H^{T}|+\left(n-q\right)\log\left(2\pi {\hat{\sigma }}^{2}\right)\right) \end{array}$$

We chose a linear basis for modeling the mean, and the squared exponential kernel (with automatic relevance determination) for the covariance function. Denoting the individual dimensions of **x** by *k* = 1…*m*, such that *x*_*ik*_ corresponds to the *k*'th dimension (e.g., *k* = 3 corresponds to τ_*out*_) of the *i*'th row of the dataset, then:


(14) $$\begin{array}{l} h{\left(x_{i}\right)}^{T}:=(1,x_{i1},\ldots ,x_{im}) \end{array}$$

(15) $$\begin{array}{l} k(x_{i},x_{j},\theta ):=\exp\left(-\frac{1}{2}\sum_{k=1}^{m}{\left|\frac{x_{ik}-x_{jk}}{\theta_{k}}\right|}^{2}\right) \end{array}$$

Defining A_*_ as the covariance matrix between prediction and training data, A_**_ as the covariance matrix between prediction data, and H_*_ as the basis matrix for predictions, then the posterior mean M and posterior variance V for predictions is given by:


(16) $$\begin{array}{l} M=H_{*}^{T}\hat{\beta }+A_{*}^{T}A_{y}^{-1}\left(\text{y}-H^{T}\hat{\beta }\right) \end{array}$$

(17) $$\begin{array}{l} V={\hat{\sigma }}^{2}(A_{**}-A_{*}^{T}A_{y}^{-1}A_{*}+{\left(H_{*}-HA_{y}^{-1}A_{*}\right)}^{T}{\left(HA_{y}^{-1}H^{T}\right)}^{-1}\\ \;\left(H_{*}-HA_{y}^{-1}A_{*}\right)) \end{array}$$

Recalling Equation (6), and noting that applying a linear operation to a Gaussian process results in a Gaussian process, then the posterior distribution for the restitution curve is also a Gaussian process, which we will refer to as a *Restitution Curve Emulator* (RCE). Reintroducing the index *c* for different principal components and defining Ψ_*C*_: = [Φ_1_(**S2**), …, Φ_*C*_(**S2**)], the RCE posterior distribution for prediction at x^*^ for *d* × 1 vector **S2** is given by:


(18) $$ℱ(x^{*},S2)\sim N(ℳ(x^{*},S2),V(x^{*},S2))$$

(19) $$ℳ(x^{*},S2)=\Phi_{0}(S2)+\Psi_{C}{\left[{ℳ}_{1}(x^{*}),\ldots ,{ℳ}_{C}(x^{*})\right]}^{T}$$

(20) $$V(x^{*},S2)=\Psi_{C}\;diag\left[V_{1}(x^{*}),\ldots ,V_{C}(x^{*})\right]\Psi_{C}^{T}$$

such that $ℳ(x^{*},S2)$ is a *d* × 1 vector and $V(x^{*},S2)$ is a *d* × *d* matrix. Note that the correlation between F values with similar *S*2 results from the principal components (*S*2 does not index the random variables). RCEs are built for ERP(S1) restitution curves in exactly the same way as for APD(S2) and CV(S2) restitution curves. Prediction with RCEs is orders of magnitude faster than simulation, with ~10^4^ predictions taking only a few seconds on a laptop (i5 gen 6 processor, 8 Gb RAM).

### 2.4. Sensitivity Analysis

Since RCEs allow probabilistic prediction of restitution curves from model parameters, they can be used to study how changes in parameters cause changes in restitution curves. RCEs are therefore ideal for exploratory model analysis. An additional advantage of the RCE approach is that *global* sensitivity analysis (SA), requiring a large number of model evaluations, can be performed across the entire parameter space. Such analysis can be performed for restitution curve values at particular S1S2 interval, e.g., APD(S2:300), but here we apply SA to the individual RCE components. The advantage to this analysis is that it is global in two different senses: (i) the SA is across the entire parameter space, rather than at a single point as for local methods; (ii) the results can be parsimoniously interpreted in terms of the effects of parameters on the *entire* restitution curve.

We use SALib (Herman and Usher, 2017) to calculate various sensitivity indices via (Saltelli's extensions to) Sobol sequences (Sobol, 2001; Saltelli, 2002; Saltelli et al., 2010), which require only model inputs (parameters) and outputs (in this case, posterior means of each RCE component). Borrowing slightly from the terminology described by SALib Toolkit, we calculate three indices: **(S1)** first-order sensitivity indices, which measure the contribution to the output variance from variation of a single parameter alone; **(S2)** second-order sensitivity indices, which measure the contribution to the output variance caused by the interaction of two parameters; (**ST**) total-effect indices, which measure the total contribution to the output variance caused by a parameter (first-order effects and all higher-order interactions). Sensitivity indices can be calculated by applying SA to posterior *samples* from the full joint posterior between all parameter values required for the Saltelli/Sobol sequence, such that the posterior variance of the emulators is accounted for and SA confidence intervals can be obtained, but we do not do that here.

### 2.5. Calibration

Given *noisy* observations **Y** from either a CV(S2) or APD(S2) restitution curve, observed for S1S2 intervals **S2_Y_**, we will assume a normal error model with homoscedastic variance $\sigma_{Y}^{2}$ linking the RCE to the observations. Although APD(S2) measurements are difficult to make, we include them here as part of our study of parameter identifiability, in order to understand whether calibration of some parameters requires APD(S2) measurements. Since measurements from S2 restitution curves involve S1 pacing across many beats in between each premature S2 beat, it is likely that errors are in fact independent, and for the purposes of investigating fundamental parameter recoverability/identifiability, a normal error model is probably a good default choice. The likelihood *p*(**Y**|**x**, σ_*Y*_) is then given by:


(21) $$\begin{array}{l} \;Y|ℱ(x,S2_{Y})\sim N(ℱ(x,S2_{Y}),\sigma_{Y}^{2}I)\\ Y\sim N(ℳ(x,S2_{Y}),V(x,S2_{Y})+\sigma_{Y}^{2}I) \end{array}$$

Measurements of ERP using an S1S2 protocol are, in fact, only observations of the S1S2 interval in which ERP lies. Representing the lower endpoint of this interval by *Y* and the interval width by Δ*S*2, the likelihood for a given parameter **x** would be $p\left(ERP\in \left(Y,Y+\Delta S2\right)|\text{x}\right)=p\left(F\left(\text{x},S2_{Y}\right)\in \left(Y,Y+\Delta S2\right)|\text{x}\right)$, which would need to be evaluated by quadrature (this likelihood would also pose difficult problems for MCMC, although this is somewhat mitigated since F is a distribution with infinite support). Rather than model *Y* = *ERP* − ϵ using ϵ ~ uniform(0, Δ*S*2), we instead model *Y* = *ERP* − δ where δ and ϵ have approximately the same distribution. We chose the following mixture of Gaussians:


(22) $$\begin{array}{l} p\left(\delta \right)=\overset{N}{\underset{i=1}{\sum }}\frac{1}{N}\frac{1}{\sqrt{2\pi s^{2}}}\exp\left(-\frac{1}{2s^{2}}{\left(\delta -m_{i}\right)}^{2}\right) \end{array}$$

where *m*_*i*_ = (*i* − 1/2)Δ*S*2 and we choose *s* = Δ*S*2/*N*. This approximates uniform(0, Δ*S*2) but has infinite support. We can then write δ as


(23) $$\begin{array}{l} \delta =Z+\overset{N}{\underset{i=1}{\sum }}I\left(K=i\right)m_{i} \end{array}$$

where $Z\sim N\left(0,s^{2}\right)$, *I* is the indicator function, and *K* is a random variable where *P*(*K* = *i*) = 1/*N* for *i* = 1…*N*. If the RCE prediction for ERP given **x** is F(x)~N(M,V) then we can write


(24) $$\begin{array}{l} Y=F\left(\text{x}\right)-Z-\overset{N}{\underset{i=1}{\sum }}I\left(K=i\right)m_{i} \end{array}$$

from which we can identify that the likelihood is


(25) $$\begin{array}{l} p\left(Y|\text{x}\right)=\overset{N}{\underset{i=1}{\sum }}\frac{1}{N}\frac{1}{\sqrt{2\pi \left(s^{2}+V\right)}}\\ \;\exp\left(-\frac{1}{2\left(s^{2}+V\right)}{\left(Y+m_{i}-M\right)}^{2}\right) \end{array}$$

Note that *Y* + *m*_*i*_ are the centers of *N* regular intervals spanning the ERP bracket. This likelihood has two main advantages for our calibration using RCEs: (1) it is analytical and requires no quadrature to be performed, as would be the case for a truncated uniform error model for ERP; (2) the distribution is continuous and has infinite support (but can be sharpened by simply adding more terms to the sum). We choose *N* = 10, which results in approximately 82% of the probability density for δ falling within the edges of the truncated uniform distribution (20 terms gives ≈ 90%, and 50 terms gives ≈ 96%), which we find works well for calibration. What is most important is that between the brackets the likelihood is virtually flat, which is what we require for ERP measured with an S1S2 protocol. Note that the *log*-likelihood, almost always utilized for optimization (and used here), should be calculated using the readily available logsumexp function, to prevent numerical underflow.

The *total* loglikelihood, accounting for measurements from different restitution curves, can be calculated by simply adding the different corresponding loglikelihoods together. Using **Y** and σ**_Y_** to represent all measurements, then the posterior distribution is given (up to a constant) by:


(26) $$\begin{array}{l} p\left(\text{x},\sigma_{\text{Y}}|\text{Y}\right)\propto p\left(\text{Y}|\text{x},\sigma_{\text{Y}}\right)p\left(\text{x}\right)p\left(\sigma_{\text{Y}}\right) \end{array}$$

We chose the prior *p*(**x**) to be truncated uniform across the same range of parameters specified in section 2.2. It is then possible to find the *maximum a posteriori* (MAP) estimate for **x** and σ_**Y**_, and also to perform Markov Chain Monte Carlo (MCMC) to obtain samples from the posterior distribution. These estimates take into account uncertainty about the observations as well as uncertainty in RCE predictions.

We use noisy measurements generated from the mMS model to demonstrate probabilistic calibration with RCEs, which also allows us to study parameter identifiability. We use CV(S2) and APD(S2) for S1: 600 ms, and ERP(S1) for S1: 400, 500, 600 ms. Since it should always be possible to collect observations for the S1 beat prior to the S2 beat, we include an observation at S2 = S1 for every S2 observation. This helps the method to learn the noise and therefore to focus on the more important question of the shape of the S2 restitution curve rather than its asymptotic limit (which can be measured much more efficiently with an S1 protocol). For simplicity of presentation and also to focus on parameter recoverability, we first obtain the MAP estimate of the parameters and the noise amplitude σ_*Y*_, and for MCMC we fix the noise amplitude to its MAP value (for calibration to real data, σ_*Y*_ should be included in MCMC in order to obtain its posterior distribution, but for studying identifiability it may be useful to fix it as done here). We perform MCMC using the Python package EMCEE (Foreman-Mackey et al., 2013), for 2,000 samples using 32 chains initialized with the MAP estimate (plus a small amount of jitter), from which we discard the first 1,000 samples as burn-in and use a thinning factor of 5.

## 3. Results

We ran the simulator from S1 350 to 700 ms at 25 ms intervals using a maximin-optimized Latin hypercube design of 500 parameters in the ranges specified in section 2.2. We discarded 166 runs where ERP(S1) was not defined for all S1, which restricted the dataset to contain ERP < 350 ms, leaving 334 simulation runs with the highest remaining ERP being 337 ms. Note that RCEs can be used to create a more careful design of parameters that produce outputs only within a desired range, but initially this is not possible since the map between the simulator inputs and outputs is not known. We restrict our analysis of S2 restitution curves to S1: 600 ms throughout, with other S1 intervals utilized for ERP(S1) only. In plots below, we denote τ_*out*_ as “Tout” etc, to assist readability.

### 3.1. Restitution Curve Emulators for CV(S2) and APD(S2)

**Figure 2:**
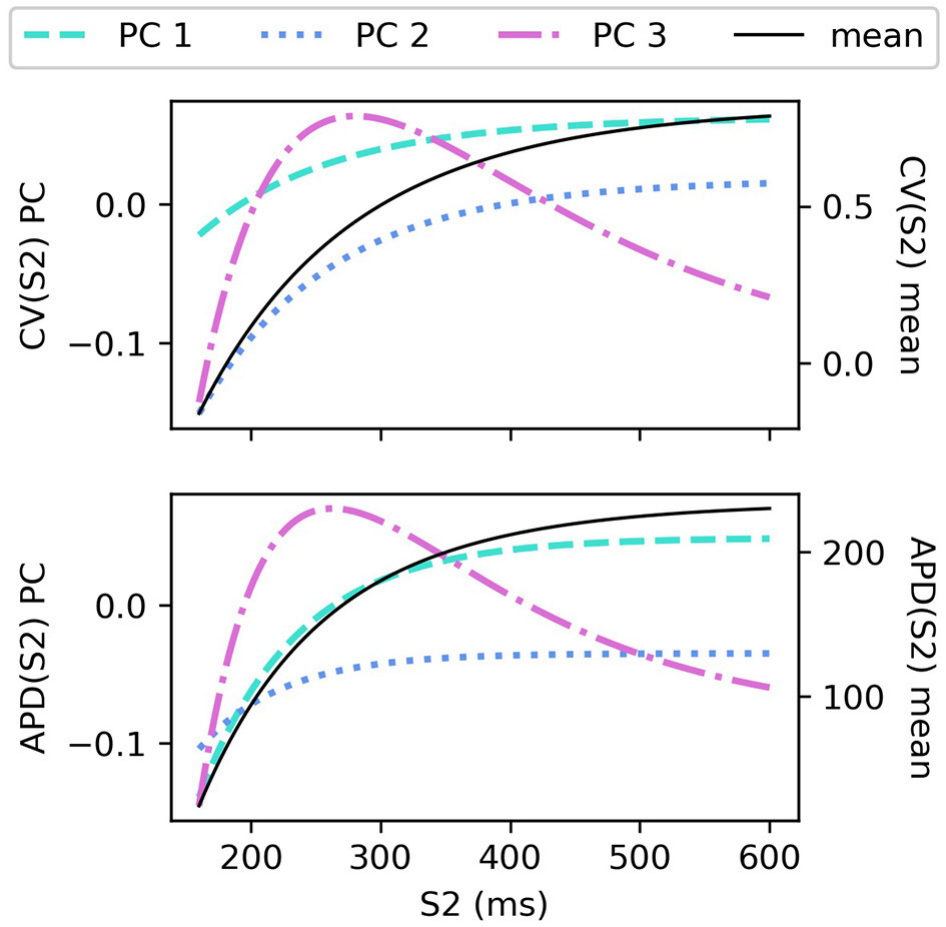
PCA components and means for CV(S2) curves and APD(S2) curves for S1: 600 ms.

The CV(S2) and APD(S2) restitution curves resampled to 1ms resolution are shown in Figure 1, colored by ERP(S1: 600) and plotted only for S1S2 intervals > ERP (the region in which observations can be collected) to aid visualization. The data means and the principal components are shown in Figure 2. We established that three principal components were sufficient to explain over 99% the variance in the dataset (of the variance retained for three components, it was divided as follows: $\Phi_{c}^{CV}$: 78.637, 20.460, 0.897%, for $\Phi_{c}^{APD}$: 68.690, 29.131, 2.148%), so we retain only three components for the RCEs. Note that there is no particular reason why a linear basis should require the same number of components as coefficients in the original non-linear mapping Equation (5), and we found that emulation of a fourth component was possible (i.e., not all higher components are just “noise”). In both cases, the first principal component, representing the direction in which the curves vary the most, represents mainly the height of the curves in the limit of long S1S2 interval, i.e., the asymptotic region. However, for CV(S2) this component is much flatter with respect to S2 than for APD(S2). For the second component the opposite is the case, showing much less variation across S2 for APD(S2) compared to CV(S2). The third components represents more subtle curvature of the “knee” of the curve, when the restitution curves begins to fall away rapidly, and is very similar for both CV(S2) and APD(S2), most notably showing the peak in approximately the same S2 location.

**Figure 3:**
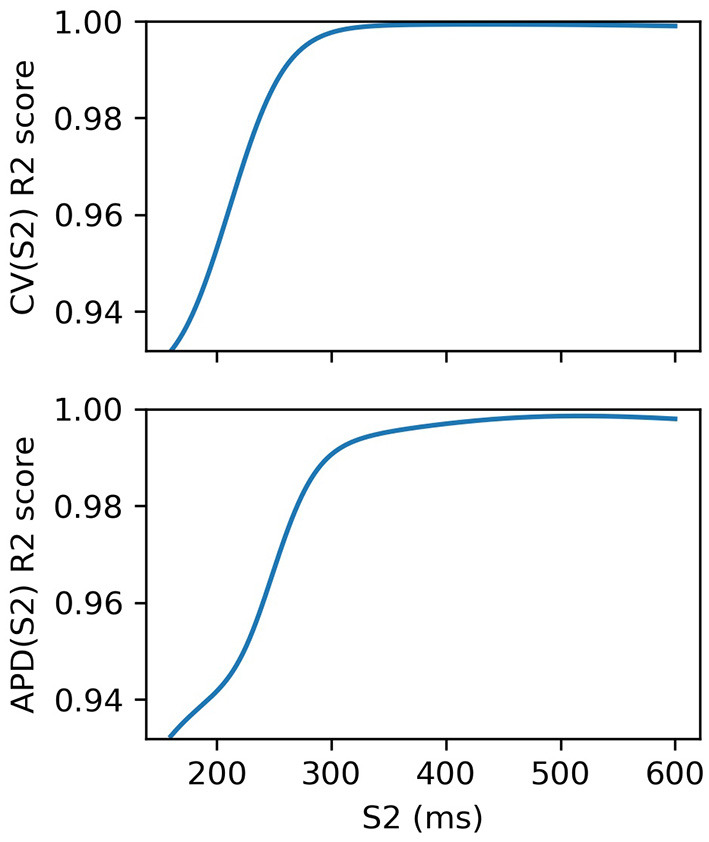
R2 scores for S2 restitution curves from 5-fold cross-validation. Performance decreases with S2, although these validation scores include RCE prediction at S2 ≤ ERP(S1) corresponding to virtual regions of the curves where no measurements can be made.

We fit an RCE for both CV(S2) and APD(S2) for S1: 600 ms. For validation, we used 5-fold cross validation and calculated the average R2 score over the folds for each S1S2 interval. The RCE validation results are shown in Figure 3, showing that the performance is extremely good, especially for long S1S2 intervals. The dependence of performance based on S1S2 interval is likely to be linked to resampling the curves into virtual regions where *S*2 ≤ *ERP*, where it is not unreasonable to suppose that the resampling itself may contain errors since resampling here is only extrapolation. RCEs do not actually need to make predictions in these regions because no measurements can be obtained here anyway. Also, RCEs predict a distribution rather than a single number. Our training dataset of 334 simulation runs was relatively small: for comparison, 3^5^ = 243 points would be required to place a data point at the corners, face centers, and body centers of a five-dimensional hypercube. In general, having validated RCEs for a particular model and range of parameters, we would consider then collecting a larger training dataset (with a more carefully chosen parameter range) for building more accurate RCEs, which would both improve accuracy and reduce posterior variance of the RCE predictions. However, we do not do this here as the validation scores are already extremely good.

**Figure 4:**
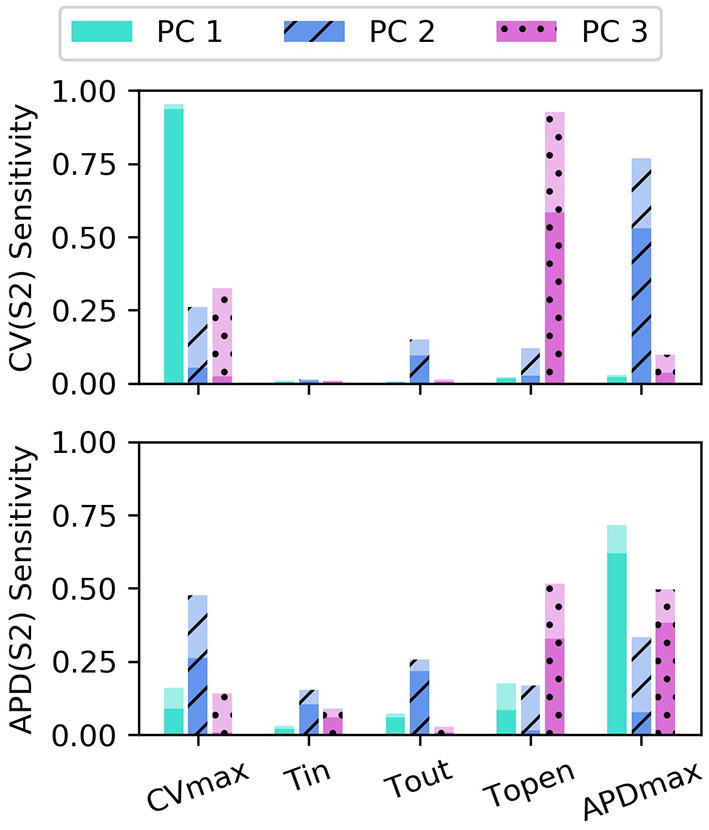
Sensitivity indices of the coordinates *f*_*c*_ of the principal components Φ_*c*_ for the model parameters for S1: 600 ms. The total-effect indices are plotted semi-transparently, with the first-order indices (which contribute to the total-effect indices) overlaid with opaque shading.

**Figure 5:**
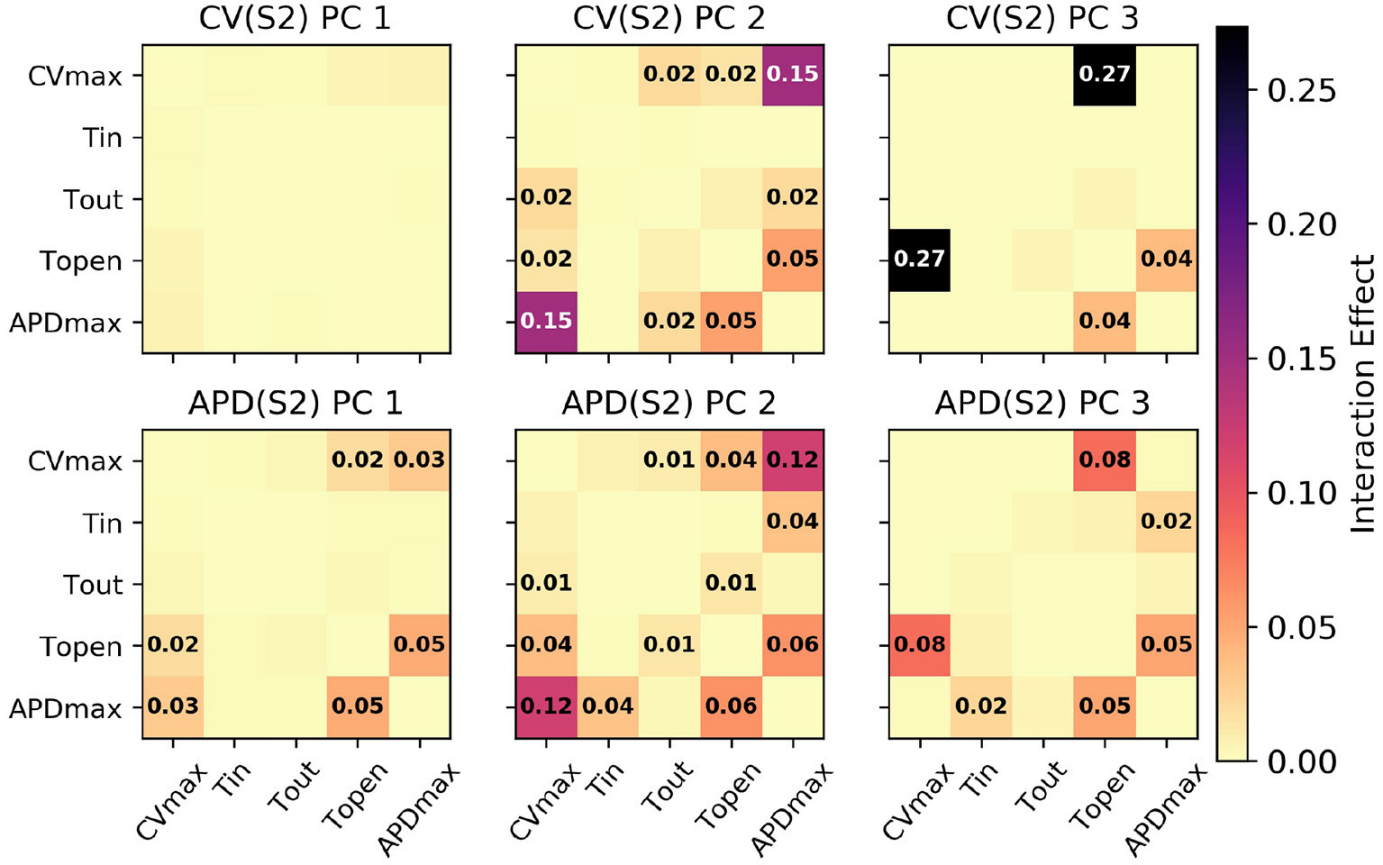
Second-order interaction effects for S1: 600 ms, with text labels applied in cells where the effects are at least 0.01 (i.e., account for at least 1% of the overall variance).

The sensitivity analyses for total-effect indices and first-order effects are shown in Figure 4, and second-order interaction effects are shown in Figure 5. In Figure 4, the total-effects are shown faded, with more opaque regions representing the first-order effects. The faded region therefore shows all higher-order effects of the parameters on the principal components. It is notable that higher-order effects are less present in the primary principal components, particularly for $\Phi_{1}^{CV}\left(S2\right)$ meaning that the asymptotic region of the restitution curve is almost entirely determined by *CV*_*max*_ as would be expected. $\Phi_{1}^{APD}$ is determined most strongly by first-order effects of τ_*out*_ and *APD*_*max*_, which can be seen to effect recovery in Equation (2) approximately for phases 2 and 3 of the action potential.

Of particular note is that $\Phi_{2}^{CV}\left(S2\right)$ is strongly effected by the same parameters that mainly determine $\Phi_{1}^{APD}\left(S2\right)$, which makes sense since $\Phi_{2}^{CV}\left(S2\right)$ mainly codes for differences between the highest and lowest values in the restitution curves, and CV takes its lowest observable values when pacing rate is close to APD. The effects of *CV*_*max*_ on $\Phi_{2}^{APD}\left(S2\right)$ are also extremely important. We had initially supposed that these effects may be artifacts, but further analysis (with a longer simulation strip, more S1 beats, and so on) revealed that this was not the case. In fact, inspection of Equation (1) reveals that such a causal effects ought to be expected: the diffusion term in Equation (1) not only depends explicitly on *D* (where $CV_{max}\propto \sqrt{D}$), but the magnitude of the diffusion term $\nabla^{2}V_{m}$ precisely depends on spatial differences which are determined by the electrical wave-front propagation velocity. This is a good example of sensitivity analysis providing insight into the model, and shows why APD restitution curves for calibration should be calculated in a tissue model rather than only from a cellular model (and since this is significantly more time consuming, is a strong motivating factor for using RCEs).

Parameter τ_*open*_ only shows first-order effects above 10% for the third principal component of both CV(S2) and APD(S2), for which it has the largest total effect of all parameters. The variance contributed by this third component to both curves is relatively small, and given that the magnitude of higher-order effects for τ_*open*_ is comparable to its first-order effects, it may be difficult to precisely calibrate τ_*open*_ using noisy measurements. Parameter τ_*in*_ shows a modest effect on $\Phi_{2}^{APD}$, likely due to the contribution to action potential duration resulting from differences in upstroke, perhaps through the same effects of electrical propagation on tissue repolarization discussed above.

Since PCA gives a linear basis, we tested using least squares to fit the basis to noisy data. This gives a Maximum Likelihood estimate under the assumption of normally distributed noise, also giving a variance measure on the fit coordinates. We had hoped that this information, considered alongside the sensitivity indices, would allow us to judge whether certain parameters were recoverable for a particular restitution curve. Unfortunately this method was not robust, often resulting in completely nonphysical restitution curves (that minimized the least squares problem, but which have zero probability i.e., cannot be produced from the simulator), and does not help to calibrate the model parameters.

### 3.2. Restitution Curve Emulators for ERP(S1)

**Figure 6:**
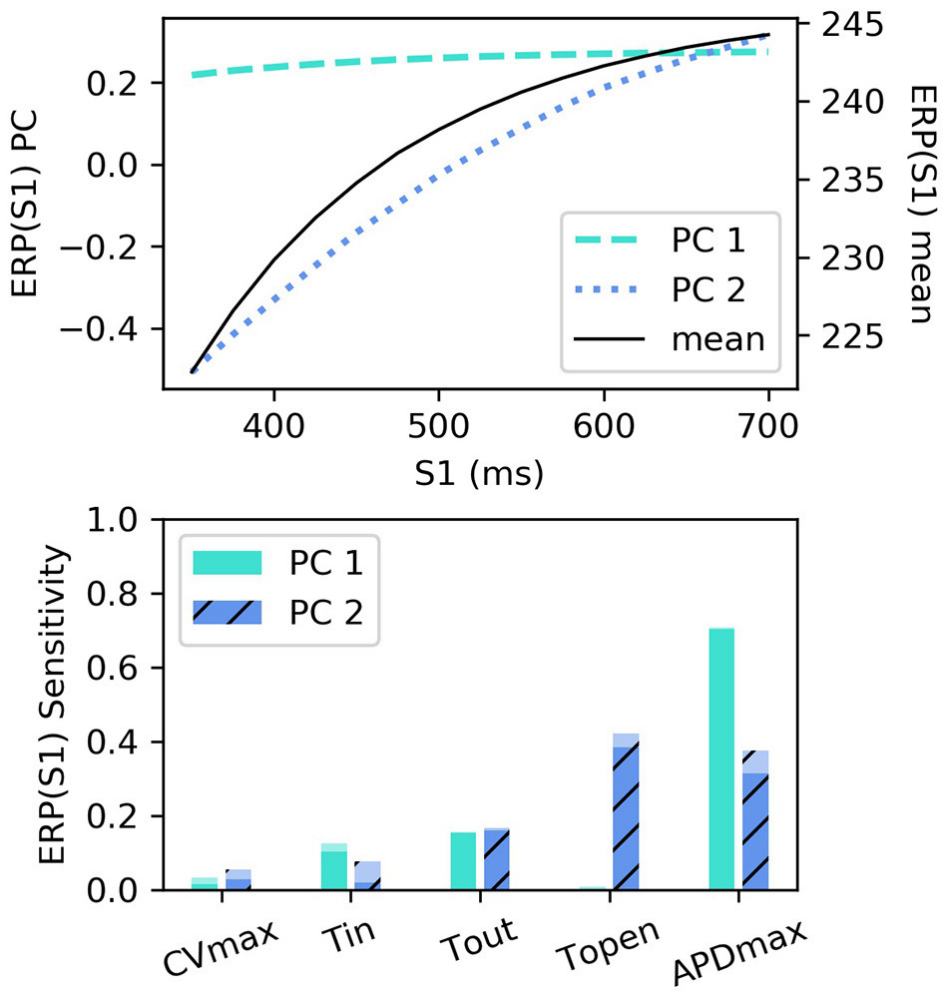
ERP(S1) restitution curves, showing (top) principal components and mean, and (bottom) first-order and total-effect sensitivity indices for the coordinates of the principal components.

RCEs were built for ERP(S1) for S1: 350–700 ms with 25 ms intervals (we did not resample these curves), using the first two principal components (the variance captured by these two components was divided 99.432 and 0.549%). The smallest R2 score was above 0.999, with little variation across S1 interval. These components are shown in Figure 6 along with the sensitivity indices [the higher-order effects are very small, so we don't show the interaction effects for ERP(S1)]. The first component almost entirely determines the height of the curve, with the lack of curvature demonstrating that the height can change very much independently of the difference between values at lowest and higher S1 interval (in other words the gradient). The second component codes mainly for the gradient of the curve (changing the difference between the lowest and highest values).

**Figure 7:**
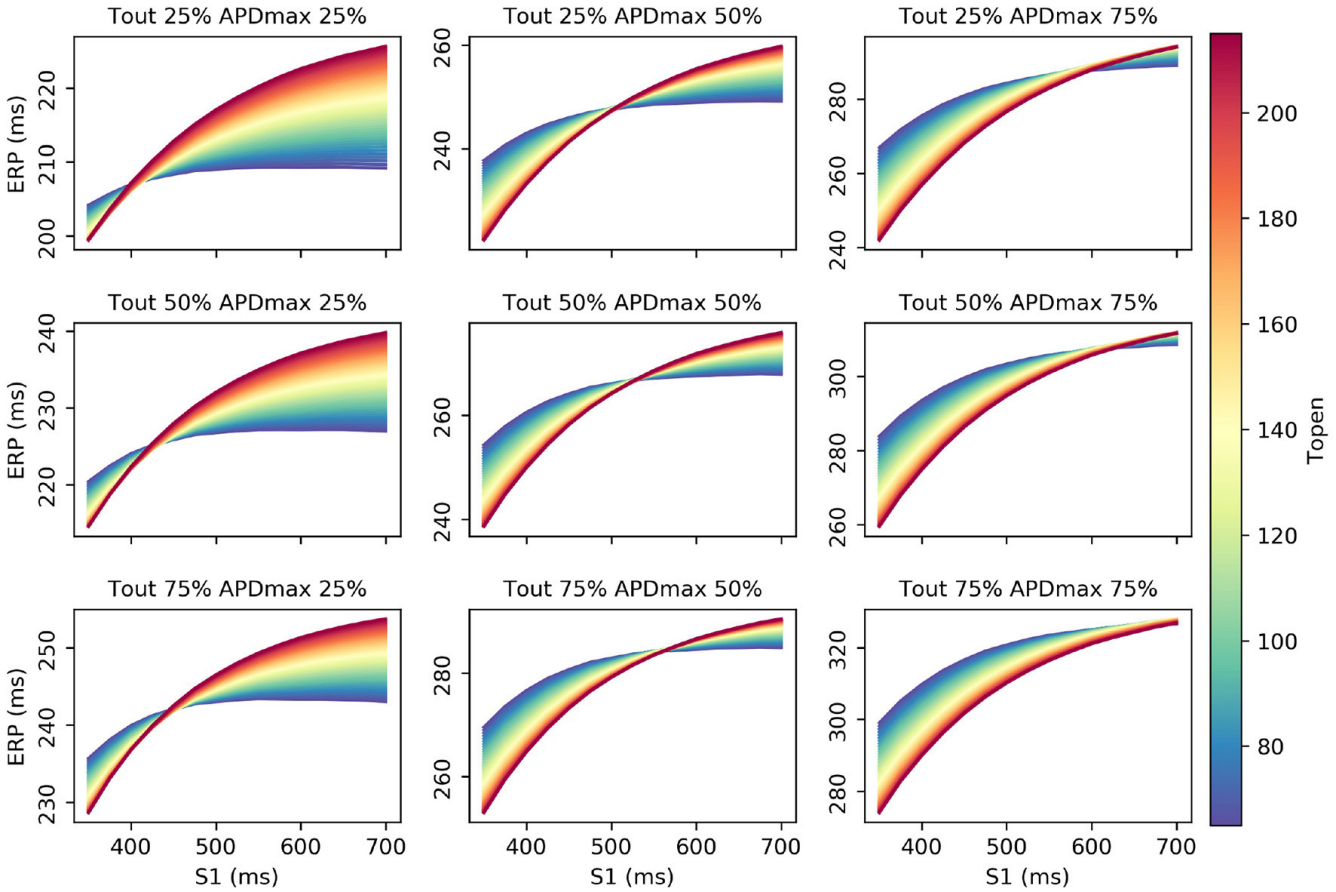
RCE predictions to explore the effects of τ_*open*_ on ERP(S1) across the parameter space.

Interestingly, τ_*open*_ has the largest first-order effect and total-effect on $\Phi_{ERP}^{2}\left(S1\right)$, though the first-order effects are similar to *APD*_*max*_ and only twice the τ_*out*_ first-order effects. Nonetheless, it is interesting to ask whether ERP(S1) observations could be used to calibrate τ_*open*_, given that it may be difficult to calibrate from noisy S2 restitution curve measurements. Figure 7 shows how RCEs can be used for exploratory model analysis, in this case visualizing the effects of particular parameters in different regions of the parameter space: we set *CV*_*max*_ and τ_*in*_ to the centers of their ranges, and each subplot corresponds to a different τ_*out*_ and *APD*_*max*_ combination, while τ_*open*_ is varied across its entire range within each subplot. These results clearly demonstrate the effects of τ_*open*_ on the ERP(S1) curves (in line with the sensitivity analysis), but show that it would be difficult to calibrate τ_*open*_ without a small resolution for the S1S2 protocol (even if the other four parameters were already known, which of course they would not be). In fact, there are regions on the ERP(S1) curve (where the curve appears to twist) where τ_*open*_ does not effect the value of the curve at all (and the S1 location of this point changes with respect to other parameters).

### 3.3. Probabilistic Calibration

To demonstrate calibration using RCEs, we rebuilt the RCEs for S1: 600 ms on 95% of the dataset, retaining 5% to use as a ground truth. From these ground truth restitution curves, we picked one for which the parameters were not too close to the edges of the parameter range so as to falsely imply a more precise calibration than is generally possible, but our results below are representative for the mMS model. We show calibration for several different combinations of measurements, explained below. A noisy dataset was generated from the ground truth restitution curves using an S1S2 interval resolution of 10 ms (this also determines the ERP resolution, as explained in section 2.5) from 170 to 360 ms for measurements [with CV and APD measurements for S2 below ERP(600) discarded], adding normally distributed noise with standard deviation 0.05*m*/*s* for CV and 5*ms* for APD. We would argue that these measurements are probably overly precise, but we chose these values to emphasize the difficulties of precise calibration even with high signal-to-noise ratio. We use ERP(S1) measurements for S1: 600, 500, 400 ms (in section 3.4, we address whether S2 restitution curves for multiple S1 are useful).

**Figure 8:**
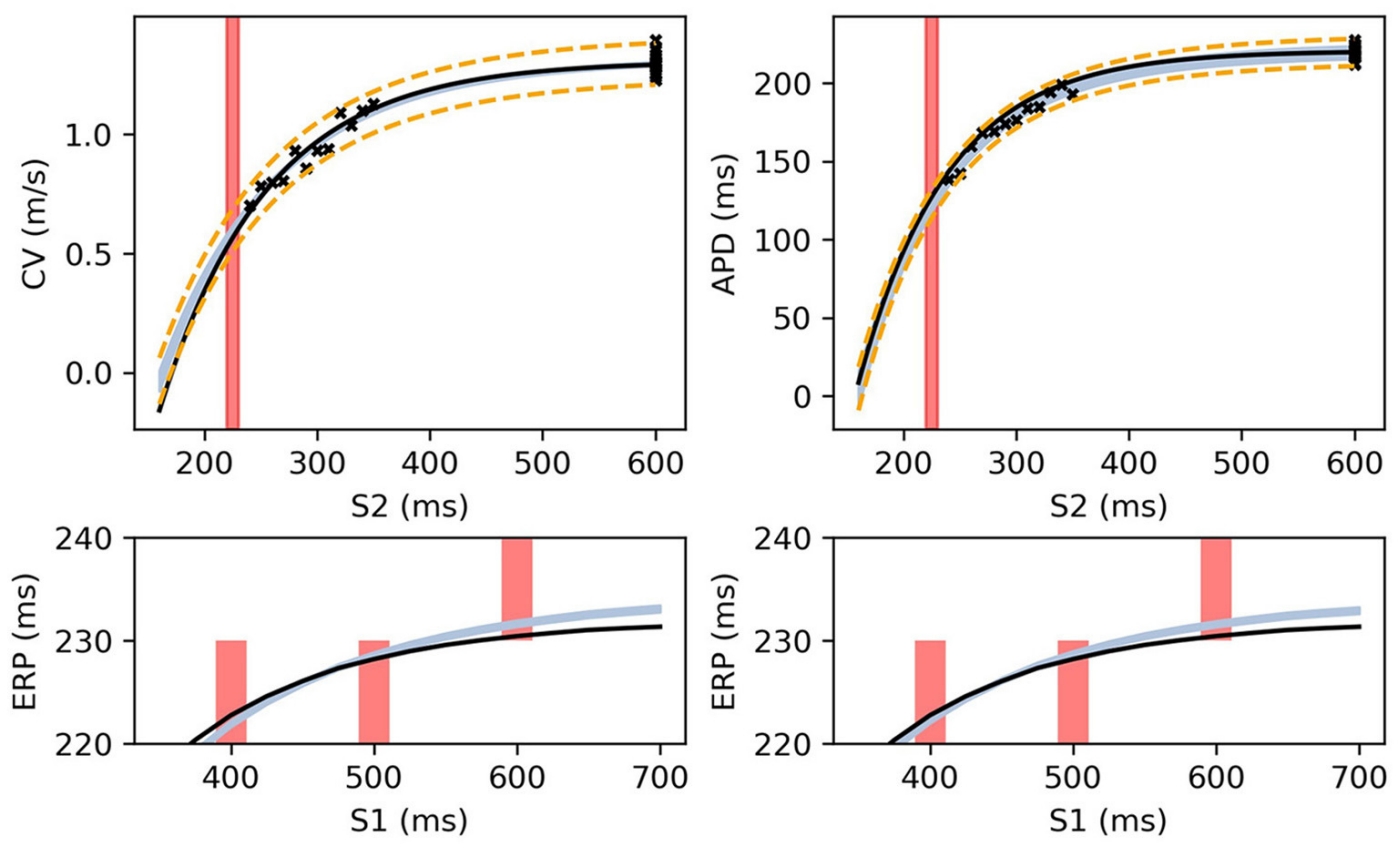
The RCE prediction from *maximum a posteriori* (MAP) parameter estimates given noisy measurements for (left) CV(S2) and ERP(S1), (right) APD(S2) and ERP(S1), shown as light shaded regions representing RCE 95% confidence intervals. The orange dashed curves show these intervals including the observation error, also learned from MAP fitting. The noisy S2 restitution data are shown as crosses, while the red shaded bars represent observed intervals containing ERP: (top): bars horizontally span ERP(S1:600) interval; (bottom) bars vertically span ERP(S1) interval for several S1. The solid black lines in all plots represent the corresponding ground truth curves.

Figure 8 shows MAP estimates of the restitution curves fitted to noisy CV(S2) and ERP(S1) data (left) and noisy APD(S2) and ERP(S1) data (right). The true restitution curves (from which the noisy observations were generated) are shown as black lines, with the cross-markers showing the noisy measurements. The ERP(S1:600) bracket (showing the S1S2 interval in which ERP is determined to lie) is plotted as a shaded interval in the S2 restitution plots, while for the ERP(S1) restitution plots thick vertical bars extend between the observed ERP brackets [the two ERP plots show the same ground truth and observed *S*2 intervals, but with different MAP fits CV (left) and APD (right)]. The posterior distribution of the RCE predictions with the MAP parameter estimates are shown as the 95% shaded confidence intervals, with the posterior mean falling exactly between these intervals but omitted for clarity. The orange dashed lines shows the confidence intervals including the estimated noise i.e., $M\pm 1.98\sqrt{V+\sigma_{Y}^{2}}$). It is clear that the MAP estimate has identified plausible restitution curves given the noisy data.

**Figure 9:**
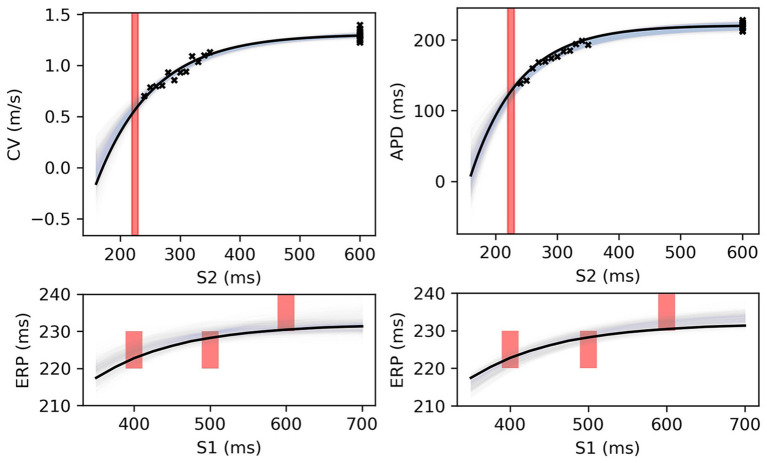
RCE predictions, shown as lightly shaded regions representing 95% confidence intervals, for 100 parameter samples from the posterior distribution given the same measurements shown in Figure 8 [black crosses are noisy S2 restitution data, red bars are observed ERP intervals, (left) MCMC with CV(S2) and ERP(S1) data, (right) MCMC with APD(S2) and ERP(S1) data].

The MAP estimates, while representing the best fits to the data, should be interpreted cautiously, as they tell us nothing about the posterior distribution for the parameters. Another random draw of noisy measurements from the same ground truth would likely result in completely different MAP estimates for the parameters. For the MCMC results for the posterior distribution below, we fix the noise σ_*Y*_ to the values obtained from the MAP estimate, in order to restrict plots and uncertainty to the model parameters (due to the S2 = S1 data, the noise was estimated extremely well, but posterior uncertainty about the noise level is generally of interest). We used MCMC to obtain samples from the posterior distribution of the parameters, as described in section 2.5, for the same data as in Figure 8. Figure 9 shows the RCE posterior means for 100 random samples from the posterior distribution obtained with MCMC. In these plots, the 95% confidence intervals have been plotted semi-transparently to assist with visualization of density. For the S2 restitution curves the density decreases away from the data, whereas for ERP(S1) restitution the density is much more uniform due to the approximately uniform error model [but will not be uniform since multiple data have been used, as opposed to data only for ERP(S1) for a single S1].

**Figure 10:**
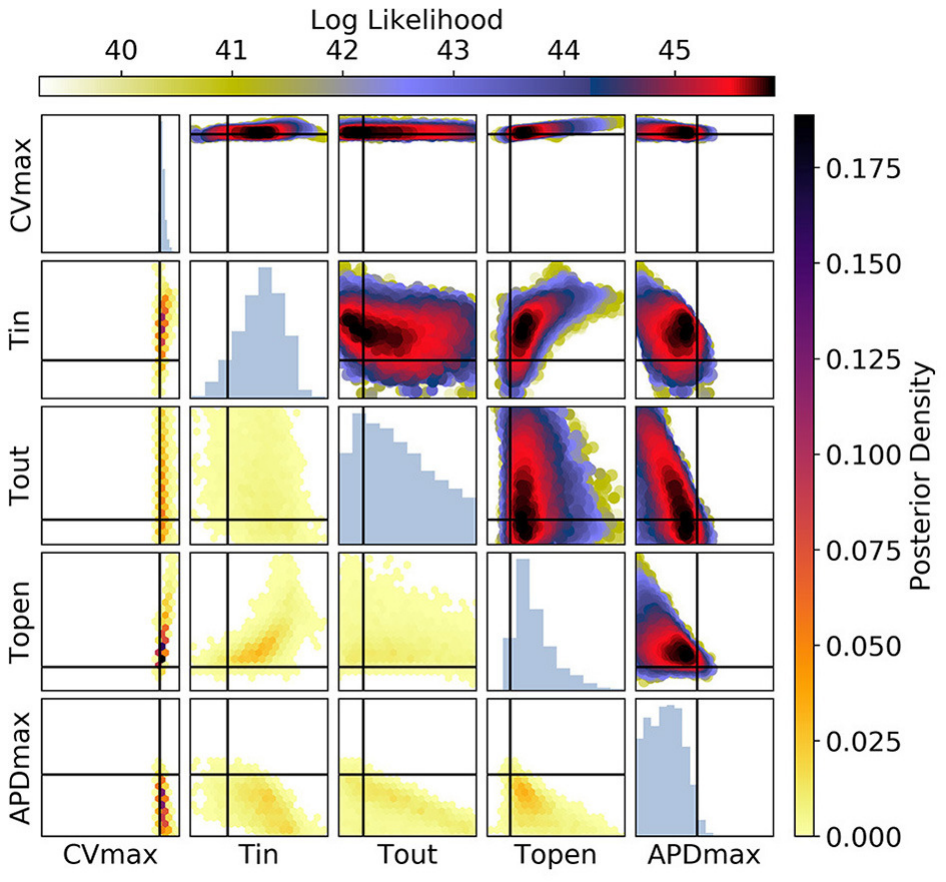
The posterior parameter distribution for fits to CV(S2) and ERP(S1) measurements. The intersection of vertical and horizontal lines mark the true parameter value. The lower diagonal shows the density via hexbin plots, while the upper diagonal shows the log likelihood values for each sample plotted in order of increasing likelihood. The diagonals show the marginal histograms of each parameter.

**Figure 11:**
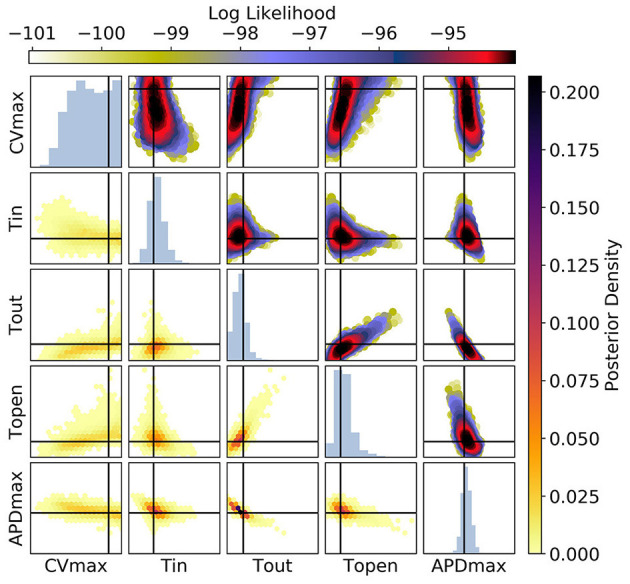
The posterior parameter distribution for fits to APD(S2) and ERP(S1) measurements. The intersection of vertical and horizontal lines mark the true parameter value. The lower diagonal shows the density via hexbin plots, while the upper diagonal shows the log-likelihood values for each sample plotted in order of increasing likelihood. The diagonals show the marginal histograms of each parameter.

Figures 10, 11 show the posterior distribution in parameter space for the data corresponding to Figure 9 (for all posterior samples after burn-in and thinning). The subplot axes span the parameter ranges given in section 2.2. Since we are presenting results for a particular ground truth curve, and the particular results will vary for every random draw of the measurement errors, we will focus on reporting the aspects of the results that are representative of the mMS model generally. However, in the Discussion we accept the difficulty of making generalizations about parameter identifiability from restitution curves. For Figure 10 [CV(S2) and ERP(S1) measurements], we see that the posterior uncertainty about all parameters except *CV*_*max*_ and τ_*open*_ is quite large (by which we mean that the marginal widths of the distribution are comparable to the parameter ranges). Generally for CV(S2) and ERP(S1) measurements, both τ_*in*_ and τ_*open*_ are quite imprecisely calibrated, but in this particular case τ_*open*_ has been calibrated fairly precisely, simply because the particular errors present in the measurements allowed for this and because the signal-to-noise ratio in this case is high because the overall value of the *CV*(*S*2) is reasonably high, allowing $\Phi_{3}^{3}$ to be learned. It can be seen for the (τ_*out*_, *APD*_*max*_) panels that these parameters appear to be constrained to a slice through parameter space, and the broad marginal histograms reflect this. This latter result could probably have been inferred from the sensitivity analysis, since these parameters both strongly influence $\Phi_{1}^{APD}$ and ERP(S1).

For Figure 11 [APD(S2) and ERP(S1) measurements], the posterior distribution is quite different to that obtained with CV(S2) and ERP(S1), although there are similarities. *CV*_*max*_ is poorly calibrated, which is not surprising in the absence of CV(S2) data. The posterior distribution is again spread as a strip through (τ_*out*_, *APD*_*max*_), indicating the difficulty of distinguishing between different contributions to APD even when the APD(S2) measurements are available. However, the peak of the posterior distribution matches the ground truth better for these parameters, which we generally find to be the case for APD(S2) and ERP(S1) measurements. Despite the ERP(S1) observations being identical to those for the CV(S2) and ERP(S1) calibration, τ_*open*_ is imprecisely calibrated here, which suggests that the precision shown in Figure 11 was the result of good estimation of $\Phi_{3}^{CV}\left(S2\right)$ rather than ERP(S1) measurements. Note that the first-order sensitivity to τ_*open*_ is almost twice as large for $\Phi_{3}^{CV}\left(S2\right)$ than for $\Phi_{3}^{APD}\left(S2\right)$, so we should expect better calibration of τ_*open*_ to CV(S2) generally. However, the signal-to-noise ratio matters a great deal, since the third principal components are relatively subtle effects.

**Figure 12:**
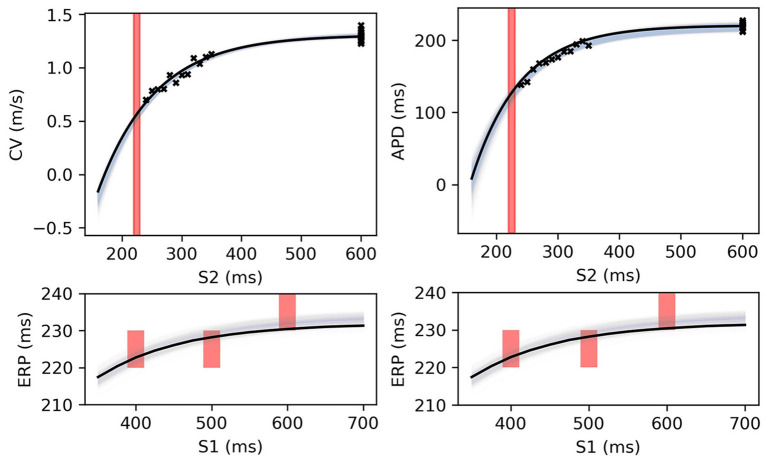
RCE predictions, shown as lightly shaded regions representing 95% confidence intervals, for 100 parameter samples from the posterior distribution given the same measurements shown in Figure 8 (black crosses are noisy S2 restitution data, red bars are observed ERP intervals). MCMC utilized CV(S2), APD(S2), and ERP(S1) data simultaneously, unlike in Figures 8, 9.

**Figure 13:**
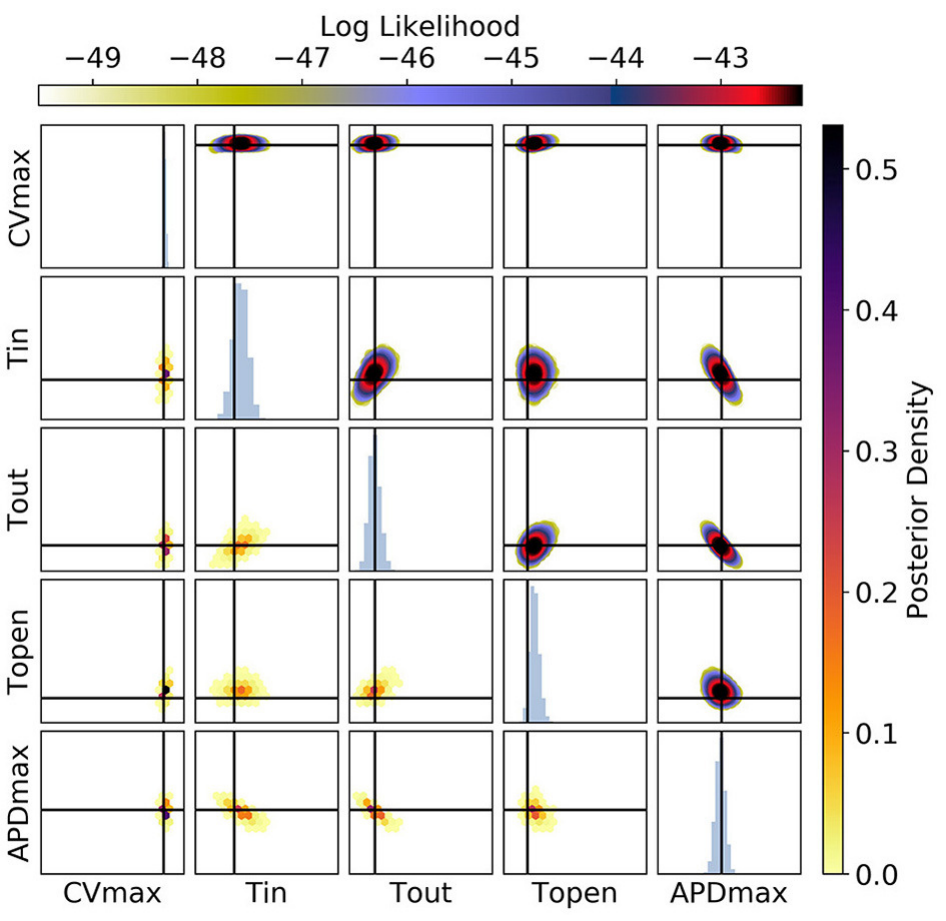
The posterior parameter distribution for calibration to CV(S2), APD(S2), and ERP(S1) measurements simultaneously. The intersection of vertical and horizontal lines mark the true parameter value. The lower diagonal shows the density via hexbin plots, while the upper diagonal shows the log likelihood values for each sample plotted in order of increasing likelihood. The diagonals show the marginal histograms of each parameter.

Figure 12 shows plots of RCE predictions for the posterior distribution obtained from MCMC using CV(S2), APD(S2), and ERP(S1) measurements simultaneously. The distribution of curves in these plots appears narrower but visually similar to Figure 9. However, Figure 13 shows that the posterior distribution of parameters is far better constrained compared to either Figure 10 or Figure 11. The peak of the distributions captures the ground truth parameter extremely well. While one reason for contraction of the posterior distribution is simply the increased amount of data, the effects are mainly down to how the data provide partially orthogonal information about the parameters. It should still be noted that these results depend highly on the particular draw of errors, and how ERP(S1) “lines up” with the intervals for S1S2 protocol resolution. Generally, we find that τ_*open*_ is the most imprecisely calibrated parameter, followed by τ_*in*_. Note that the shape of the posterior distribution across (τ_*out*_, *APD*_*max*_) is still strip shaped.

### 3.4. Restitution Surfaces

**Figure 14:**
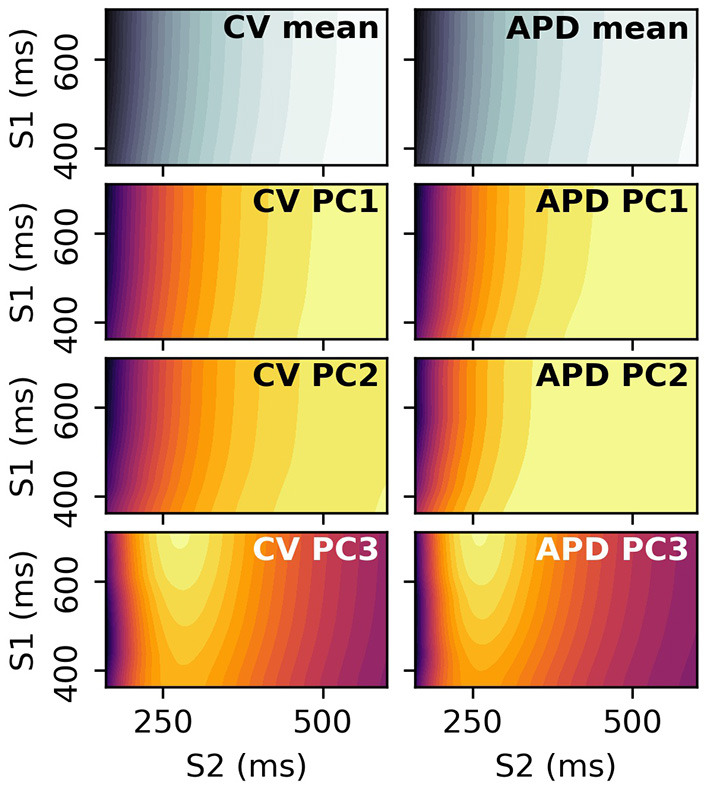
Principal components and means of CV(S1,S2) and APD(S1,S2) restitution surfaces, displayed as contour plots where dark/light colors represent low/high values, respectively. These surfaces are 2D analogues to the curves in Figure 2.

S2 restitution curves can be obtained for a range of S1 values, and the resulting data arranged into a 2D space of S1 and S2 to give *restitution surfaces*. Each S1S2 combination corresponds to a dimension in the output space, and PCA can be performed on these 2D images. The resulting principal components can be visualized by plotting the elements of the principal components against their corresponding S1 and S1S2 interval. Figure 14 shows the mean and first three principal components of the CV(S1,S2) and APD(S1,S2) restitution surfaces, plotted as contours in order to help with visualization (the colorbars are not shown as they are not required for our discussion). RCEs could be built with these principal components (such emulators might be called Restitution Surface Emulators) such that the surfaces could be predicted from the parameters.

Figure 14 shows that the principal components vary relatively little with S1 interval, since the contour lines are nearly parallel to the S1 axis. This means that the restitution surfaces are highly correlated with S1 interval. For the third component around S1S2 intervals of 275 ms, the peak in the restitution curve seen in the S2 restitution curve is now a ridge in the restitution surface, decreasing in height with decreasing S1 interval. These images show that collecting restitution curves for e.g., CV(S1: 400 ms) will be very similar to CV(S1: 600 ms, S2: 400 ms) etc., such that S1 pacing could be used to collect similar data more efficiently with S1 pacing rather than an S1S2 protocol, for values of S1 interval for which steady pacing is possible. However, learning the principal modes of variation of the surfaces (as with the curves) requires measurements at S1S2 interval far below values of S1 interval that can be used for steady pacing in the clinical setting, so it is not clear that such measurements would be useful. Furthermore, restitution curves for higher S1 interval have a larger variation in values over S2 (even only considering values for *S*2 < 350*ms*, as can be seen in the third component in particular), making calibration with noisy data more robust for higher S1 as the signal-to-noise ratio will be higher (equivalently, differentiation between different curves is easier). Upper values of S1 interval are limited by the heart's own natural pacemaker behavior, so S1: 600 ms is probably a conservative choice for clinical pacing. In summary, it is probably not worth collecting restitution data for a variety of S1 values, except when it is obtained for free due to pacing at different S1 intervals to obtain ERP(S1).

### 3.5. Diastolic Interval Restitution Curves

We also investigated RCEs of Diastolic Interval (DI) restitution curves, where *DI* = *S*2 − *ERP*, such that all curves begin at *DI* = 0, which seem advantageous since the resampling would not produce any “virtual” regions (as for S2 restitutions for S1S2 interval < ERP). The worst effect that these virtual regions can have for S2 restitution is that PCA will account for variation between curves that considers these “virtual” regions, so the dimensionality reduction for DI curves may be slightly more optimal than for S2 curves (although there may be errors in DI curves caused by a finite ERP resolution). Alternative restitution curve fits that are asymptotic for *S*2 < *ERP* (such as a sigmoid curve, which fits the restitution curves from many electrophysiology models) might reduce these effects from the virtual region. However, it is trivial to simply increase the number of principal components in RCEs if required. S2 restitution curves can be calibrated to data with and without ERP measurements, but this is not the case for DI restitution curves, since the assignment of DI to the measured data requires predicting ERP. This makes calibration highly dependent on ERP prediction, but in a purely artificial way caused by the way the problem is posed. Furthermore, given that RCEs predict a distribution, the likelihood calculations would involve a convolution, or “blurring,” of predictions across DI, since the DI “label” of the data would have a distribution. Since these difficulties are completely avoided by simply using S2 restitution curves, we do not currently see any benefit to emulating DI restitution curves.

## 4. Discussion

In the present study, we have demonstrated a way to emulate restitution curves by using Gaussian processes to predict the principal component coordinates of restitution curves from model parameters. These Restitution Curve Emulators (RCEs) make it possible to rapidly and accurately predict CV, APD, and ERP restitution curves from model parameters, allowing for sensitivity analysis, model exploration, and Bayesian calibration to noisy data. We also developed an analytical likelihood function for ERP observations, which is especially useful for calibration with RCEs. The main benefits of RCEs are prediction speed and quantification of prediction uncertainty, but an additional advantage is their parsimonious structure: sensitivity analysis can be performed for the separate principal components, and the problem of recoverability can be interpreted as the problem of learning features of restitution curves that are sensitive to changes in parameters.

It is difficult to guess what combination of measurements will be required to identify model parameters. Larger first-order sensitivity indices for more primary features suggest higher identifiability, and if several parameters have similar effects on a feature then it will be difficult to distinguish them from data about that feature alone. However, it is difficult to make general statements about identifiability/recoverability of parameters given a pacing protocol: it may turn out that parameters are recoverable in some parts of parameter space but not others, or that calibration is extremely sensitive to measurement errors, or that pacing resolution does not allow to resolve different restitution curves effectively. It is even quite difficult to generalize about how the credible intervals in the posterior distribution depend on the noise levels/pacing resolution in the data, although RCEs could be used to empirically determine this relation via brute force sampling throughout the parameter space. RCEs could find application in the design of clinical data collection protocols intended for the calibration of personalized models.

The identifiability of model parameters, as well as the practical consideration of whether parameters can be recovered from sparse and noisy clinical data, remain challenging issues (Whittaker et al., 2020) even with the mMS model, which can be considered a minimal model. It remains to be seen if more detailed models that have been designed to minimize the number of free parameters can overcome these obstacles (Pathmanathan et al., 2019). Model discrepancy can be an issue with calibrating models, often manifesting as an inability to simultaneously reproduce two behaviors (Coveney and Clayton, 2018; Lawson et al., 2018). In our framework, the error variance can include variance from noise as well as discrepancy variance (Vernon et al., 2010), but more complex modeling of discrepancy would also be possible (Brynjarsdóttir and O'Hagan, 2014), such as modeling systematic offset using a bias term in the likelihood.

Extending our approach to biophysically detailed cell models is a logical next step, which could be used either to examine the properties of these models in detail, and to examine parameter recoverability for simulated clinical measurements. Since our approach involves emulating the principal components of restitution curves, we expect that it can be extended to more detailed models without incurring computational costs apart from those involved in computing the initial set of restitution curves. Another option is the use of more complex stimulation protocols (Groenendaal et al., 2015; Beattie et al., 2018), which can work well for the experimental setting but could be difficult to deploy clinically.

Posterior uncertainty in calibration for model personalization should not be overlooked, as it is important that uncertainty is propagated forward to predictions when personalized models are used for diagnosis or decision support in the clinical setting. Calibration methods that obtain parameterizations consistent with observations but without obtaining the posterior distribution, and especially methods that provide only a single fit to the data, are not well-suited to this task. The methods presented in this paper were motivated by the need to perform probabilistic calibration with clinical data such as restitution curves. We suggest that the English idiom “*How long is a piece of string?”*, used to reply to questions that require an answer to be calculated on a case-by-case basis, be used as a rule-of-thumb when considering questions about the identifiability of electrophysiology model parameters from restitution curve measurements. We believe the answer requires calculating the posterior distribution of the model parameters given the data, and that RCEs are an extremely effective tool with which to do this.

## Data Availability Statement

The raw data supporting the conclusions of this article will be made available by the authors, without undue reservation.

## Author Contributions

SC conceived the study and methods, wrote the simulation, emulation, analysis code, and prepared the majority of the manuscript. CC advised on CARP simulations. JO and RW advised on emulation, sensitivity analysis, and calibration. SN advised on clinical applications. RC oversaw the project and assisted with manuscript preparation. All authors contributed to the article and approved the submitted version.

## Conflict of Interest

The authors declare that the research was conducted in the absence of any commercial or financial relationships that could be construed as a potential conflict of interest.

## References

[B1] Beattie K. A. Hill A. P. Bardenet R. Cui Y. Vandenberg J. I. Gavaghan D. J.. (2018). Sinusoidal voltage protocols for rapid characterisation of ion channel kinetics. J. Physiol. 596, 1813–1828. 10.1113/JP275733 29573276 PMC5978315

[B2] Boyle P. M. Ochs A. R. Ali R. L. Paliwal N. Trayanova N. A. (2021). Characterizing the arrhythmogenic substrate in personalized models of atrial fibrillation: sensitivity to mesh resolution and pacing protocol in AF models. EP Europace 23(Suppl. 1), i3–i11. 10.1093/europace/euaa385 33751074 PMC7943367

[B3] Brynjarsdóttir J. O'Hagan A. (2014). Learning about physical parameters: the importance of model discrepancy. Inverse Probl. 30:114007. 10.1088/0266-5611/30/11/114007

[B4] Cairns D. I. Fenton F. H. Cherry E. M. (2017). Efficient parameterization of cardiac action potential models using a genetic algorithm. Chaos 27:093922. 10.1063/1.5000354 28964158

[B5] Chang E. T. Y. Strong M. Clayton R. H. (2015). Bayesian sensitivity analysis of a cardiac cell model using a Gaussian process emulator. PLoS ONE 10:e0130252. 10.1371/journal.pone.0130252 26114610 PMC4482712

[B6] Cherry E. M. Evans S. J. (2008). Properties of two human atrial cell models in tissue: restitution, memory, propagation, and reentry. J. Theor. Biol. 254, 674–690. 10.1016/j.jtbi.2008.06.030 18652834 PMC2630028

[B7] Conti S. O'Hagan A. (2010). Bayesian emulation of complex multi-output and dynamic computer models. J. Stat. Plann. Inference 140, 640–651. 10.1016/j.jspi.2009.08.006

[B8] Corrado C. Niederer S. A. (2016). A two-variable model robust to pacemaker behaviour for the dynamics of the cardiac action potential. Math. Biosci. 281, 46–54. 10.1016/j.mbs.2016.08.010 27590776 PMC5082966

[B9] Corrado C. Whitaker J. Chubb H. Williams S. Wright M. Gill J.. (2017). Personalized models of human atrial electrophysiology derived from endocardial electrograms. IEEE Trans. Biomed. Eng. 64, 735–742. 10.1109/TBME.2016.2574619 28207381

[B10] Coveney S. Clayton R. H. (2018). Fitting two human atrial cell models to experimental data using Bayesian history matching. Prog. Biophys. Mol. Biol. 139, 43–58. 10.1016/j.pbiomolbio.2018.08.001 30145156

[B11] Coveney S. Clayton R. H. (2020). Sensitivity and uncertainty analysis of two human atrial cardiac cell models using Gaussian process emulators. Front. Physiol. 11:364. 10.3389/fphys.2020.00364 32390867 PMC7191317

[B12] Coveney S. Corrado C. Roney C. H. O'Hare D. Williams S. E. O'Neill M. D.. (2020). Gaussian process manifold interpolation for probabilistic atrial activation maps and uncertain conduction velocity. Philos. Trans. R. Soc. A Math. Phys. Eng. Sci. 378:20190345. 10.1098/rsta.2019.0345 32448072 PMC7287339

[B13] Dhamala J. Bajracharya P. Arevalo H. J. Sapp J. Horácek B. M. Wu K. C.. (2020). Embedding high-dimensional bayesian optimization via generative modeling: parameter personalization of cardiac electrophysiological models. Med. Image Anal. 62:101670. 10.1016/j.media.2020.101670 32171168 PMC7237332

[B14] Dokos S. Lovell N. H. (2004). Parameter estimation in cardiac ionic models. Prog. Biophys. Mol. Biol. 85, 407–431. 10.1016/j.pbiomolbio.2004.02.002 15142755

[B15] Fenton F. Karma A. (1998). Fiber-rotation-induced vortex turbulence in thick myocardium. Phys. Rev. Lett. 81, 481–484. 10.1103/PhysRevLett.81.481

[B16] Fenton F. H. Cherry E. M. Hastings H. M. Evans S. J. (2002). Multiple mechanisms of spiral wave breakup in a model of cardiac electrical activity. Chaos 12, 852–892. 10.1063/1.1504242 12779613

[B17] Fink M. Niederer S. A. Cherry E. M. Fenton F. H. Koivumaki J. T. Seemann G.. (2011). Cardiac cell modelling: observations from the heart of the cardiac physiome project. Prog. Biophys. Mol. Biol. 104, 2–21. 10.1016/j.pbiomolbio.2010.03.002 20303361

[B18] Foreman-Mackey D. Hogg D. W. Lang D. Goodman J. (2013). emcee: The MCMC hammer. Publ. Astron. Soc. Pac. 125, 306–312. 10.1086/670067

[B19] Groenendaal W. Ortega F. A. Kherlopian A. R. Zygmunt A. C. Krogh-Madsen T. Christini D. J. (2015). Cell-specific cardiac electrophysiology models. PLoS Comput. Biol. 11:e1004242. 10.1371/journal.pcbi.1004242 25928268 PMC4415772

[B20] Herman J. Usher W. (2017). SALib: An open-source python library for sensitivity analysis. J. Open Source Softw. 2:97. 10.21105/joss.00097

[B21] Higdon D. Gattiker J. Williams B. Rightley M. (2008). Computer model calibration using high-dimensional output. J. Am. Stat. Assoc. 103, 570–583. 10.1198/016214507000000888

[B22] Johnstone R. H. Chang E. T. Bardenet R. de Boer T. P. Gavaghan D. J. Pathmanathan P.. (2016). Uncertainty and variability in models of the cardiac action potential: can we build trustworthy models? J. Mol. Cell. Cardiol. 96, 49–62. 10.1016/j.yjmcc.2015.11.018 26611884 PMC4915860

[B23] Konukoglu E. Relan J. Cilingir U. Menze B. H. Chinchapatnam P. Jadidi A.. (2011). Efficient probabilistic model personalization integrating uncertainty on data and parameters: application to Eikonal-Diffusion models in cardiac electrophysiology. Prog. Biophys. Mol. Biol. 107, 134–146. 10.1016/j.pbiomolbio.2011.07.002 21763715

[B24] Krogh-Madsen T. Sobie E. A. Christini D. J. (2016). Improving cardiomyocyte model fidelity and utility via dynamic electrophysiology protocols and optimization algorithms. J. Physiol. 594, 2525–2536. 10.1113/JP270618 26661516 PMC4850194

[B25] Lawson B. A. J. Drovandi C. C. Cusimano N. Burrage P. Rodriguez B. Burrage K. (2018). Unlocking datasets by calibrating populations of models to data density: a study in atrial electrophysiology. Sci. Adv. 4:e1701676. 10.1126/sciadv.1701676 29349296 PMC5770172

[B26] Lawson B. A. J. Oliveira R. S. Berg L. A. Silva P. A. A. Burrage K. dos Santos R. W. (2020). Variability in electrophysiological properties and conducting obstacles controls re-entry risk in heterogeneous ischaemic tissue. Philos. Trans. R. Soc. A Math. Phys. Eng. Sci. 378:20190341. 10.1098/rsta.2019.0341 32448068 PMC7287337

[B27] Loewe A. Wilhelms M. Schmid J. Krause M. J. Fischer F. Thomas D.. (2015). Parameter estimation of ion current formulations requires hybrid optimization approach to be both accurate and reliable. Front. Bioeng. Biotechnol. 3:209. 10.3389/fbioe.2015.00209 26793704 PMC4710757

[B28] Longobardi S. Lewalle A. Coveney S. Sjaastad I. Espe E. K. S. Louch W. E.. (2020). Predicting left ventricular contractile function via gaussian process emulation in aortic-banded rats. Philos. Trans. R. Soc. A Math. Phys. Eng. Sci. 378:20190334. 10.1098/rsta.2019.0334 32448071 PMC7287330

[B29] Mitchell C. Schaeffer D. (2003). A two-current model for the dynamics of cardiac membrane. Bull. Math. Biol. 65, 767–793. 10.1016/S0092-8240(03)00041-7 12909250

[B30] Muszkiewicz A. Britton O. J. Gemmell P. Passini E. Carlos S. Zhou X.. (2015). Variability in cardiac electrophysiology : Using experimentally- calibrated populations of models to move beyond the single virtual physiological human paradigm. Prog. Biophys. Mol. Biol. 120, 115–127. 10.1016/j.pbiomolbio.2015.12.002 26701222 PMC4821179

[B31] Niederer S. A. Lumens J. Trayanova N. A. (2019). Computational models in cardiology. Nat. Rev. Cardiol. 16, 100–111. 10.1038/s41569-018-0104-y 30361497 PMC6556062

[B32] Oakley J. (1999).Oakley J. (1999). Bayesian Uncertainty Analysis for Complex Computer Codes. University of Sheffield, 143.

[B33] Pathmanathan P. Cordeiro J. M. Gray R. A. (2019). Comprehensive uncertainty quantification and sensitivity analysis for cardiac action potential models. Front. Physiol. 10:721. 10.3389/fphys.2019.00721 31297060 PMC6607060

[B34] Plank G. Loewe A. Neic A. Augustin C. Huang Y.-L. Gsell M. A.. (2021). The openCARP simulation environment for cardiac electrophysiology. Comput. Methods Prog. Biomed. 208:106223. 10.1016/j.cmpb.2021.106223 34171774

[B35] Rasmussen C. E. Williams C. K. I. (2006). Gaussian Processes for Machine Learning, 1st Edn. Cambridge: MIT Press. 10.7551/mitpress/3206.001.0001

[B36] Relan J. Chinchapatnam P. Sermesant M. Rhode K. Delingette H. Razavi R.. (2010). “Coupled personalisation of electrophysiology models for simulation of induced ischemic ventricular tachycardia,” in Medical Image Computing and Computer-Assisted Intervention-MICCAI 2010, Vol. 6362, eds Hutchison D. Kanade T. Kittler J. Kleinberg J. M. Mattern F. Mitchell J. C. Naor M. Nierstrasz O. Pandu Rangan C. Steffen B. Sudan M. Terzopoulos D. Tygar D. Vardi M. Y. Weikum G. Jiang T. Navab N. Pluim J. P. W. Viergever M. A. (Berlin; Heidelberg: Springer Berlin Heidelberg), 420–428. 10.1007/978-3-642-15745-5_52 20879343

[B37] Relan J. Pop M. Delingette H. Wright G. A. Ayache N. Sermesant M. (2011). Personalization of a cardiac electrophysiology model using optical mapping and MRI for prediction of changes with pacing. IEEE Trans. Biomed. Eng. 58, 3339–3349. 10.1109/TBME.2011.2107513 21257368

[B38] Saltelli A. (2002). Making best use of model evaluations to compute sensitivity indices. Comput. Phys. Commun. 145, 280–297. 10.1016/S0010-4655(02)00280-1

[B39] Saltelli A. Annoni P. Azzini I. Campolongo F. Ratto M. Tarantola S. (2010). Variance based sensitivity analysis of model output. Design and estimator for the total sensitivity index. Comput. Phys. Commun. 181, 259–270. 10.1016/j.cpc.2009.09.018

[B40] Sarkar A. X. Sobie E. A. (2010). Regression analysis for constraining free parameters in electrophysiological models of cardiac cells. PLoS Comput. Biol. 6:e1000914. 10.1371/journal.pcbi.1000914 20824123 PMC2932676

[B41] Sermesant M. Chabiniok R. Chinchapatnam P. Mansi T. Billet F. Moireau P.. (2012). Patient-specific electromechanical models of the heart for the prediction of pacing acute effects in CRT: a preliminary clinical validation. Med. Image Anal. 16, 201–215. 10.1016/j.media.2011.07.003 21920797

[B42] Smirnov D. Pikunov A. Syunyaev R. Deviatiiarov R. Gusev O. Aras K.. (2020). Genetic algorithm-based personalized models of human cardiac action potential. PLoS ONE 15:e244687. 10.1371/journal.pone.0244687 32392258 PMC7213718

[B43] Sobol I. (2001). Global sensitivity indices for nonlinear mathematical models and their monte carlo estimates. Math. Comput. Simul. 55, 271–280. 10.1016/S0378-4754(00)00270-6

[B44] Tixier E. Lombardi D. Rodriguez B. Gerbeau J.-F. (2017). Modelling variability in cardiac electrophysiology: a moment-matching approach. J. R. Soc. Interface 14:20170238. 10.1098/rsif.2017.0238 28835541 PMC5582121

[B45] Vernon I. Goldstein M. Bower R. G. (2010). Galaxy formation: a Bayesian uncertainty analysis. Bayesian Anal. 5, 619–669. 10.1214/10-BA524

[B46] Whittaker D. G. Clerx M. Lei C. L. Christini D. J. Mirams G. R. (2020). Calibration of ionic and cellular cardiac electrophysiology models. WIREs Syst. Biol. Med. 12:e1482. 10.1002/wsbm.1482 32084308 PMC8614115

[B47] Wilkinson R. D. (2010). “Chapter 10: Bayesian calibration of expensive multivariate computer experiments,” in Large-Scale Inverse Problems and Quantification of Uncertainty, eds Biegler L. Biros G. Ghattas O. Heinkenschloss M. Keyes D. E. Mallick B. Marzouk Y. Tenorio L. van Bloemen Waanders B. Willcox K. (John Wiley & Sons, Ltd.), 195–215. 10.1002/9780470685853.ch10

