# Corrigendum: Bayesian Calibration of Electrophysiology Models Using Restitution Curve Emulators

**DOI:** 10.3389/fphys.2021.765622

**Published:** 2021-10-04

**Authors:** Sam Coveney, Cesare Corrado, Jeremy E. Oakley, Richard D. Wilkinson, Steven A. Niederer, Richard H. Clayton

**Affiliations:** ^1^Insigneo Institute for In-Silico Medicine and Department of Computer Science, University of Sheffield, Sheffield, United Kingdom; ^2^Division of Imaging Sciences and Biomedical Engineering, King's College London, London, United Kingdom; ^3^School of Mathematics and Statistics, University of Sheffield, Sheffield, United Kingdom; ^4^School of Mathematical Sciences, University of Nottingham, Nottingham, United Kingdom

**Keywords:** restitution, electrophysiology, cardiology, Gaussian processes, emulation, sensitivity analysis, calibration, Bayesian

In the original article, there was an omission. Equations for the posterior distribution of Restitution Curve Emulators for prediction at multiple *S*2 values were not provided, but these equations are required in Equation (21). Equations (18)–(20) should have been generalized from scalar *S*2 to vector **S2**.

A correction has been made to the last paragraph of Section 2. Methods, Sub-section 2.3 Restitution Curve Emulators:

Recalling Equation (6), and noting that applying a linear operation to a Gaussian process results in a Gaussian process, then the posterior distribution for the restitution curve is also a Gaussian process, which we will refer to as a *Restitution Curve Emulator* (RCE). Reintroducing the index *c* for different principal components and defining Ψ_*C*_: = [Φ_1_(**S2**), …, Φ_*C*_(**S2**)], the RCE posterior distribution for prediction at x^*^ for *d* × 1 vector **S2** is given by:


(18) $$ℱ(x^{*},S2)\sim N(ℳ(x^{*},S2),V(x^{*},S2))$$

(19) $$ℳ(x^{*},S2)=\Phi_{0}(S2)+\Psi_{C}{\left[{ℳ}_{1}(x^{*}),\ldots ,{ℳ}_{C}(x^{*})\right]}^{T}$$

(20) $$V(x^{*},S2)=\Psi_{C}\;diag\left[V_{1}(x^{*}),\ldots ,V_{C}(x^{*})\right]\Psi_{C}^{T}$$

such that $ℳ(x^{*},S2)$ is a *d* × 1 vector and $V(x^{*},S2)$ is a *d* × *d* matrix. Note that the correlation between F values with similar *S*2 results from the principal components (*S*2 does not index the random variables). RCEs are built for ERP(S1) restitution curves in exactly the same way as for APD(S2) and CV(S2) restitution curves. Prediction with RCEs is orders of magnitude faster than simulation, with ~10^4^ predictions taking only a few seconds on a laptop (i5 gen 6 processor, 8 Gb RAM).

In the original article, there was an omission. Equation (21) was missing an identity matrix factor.

A correction has been made to Section 2. Methods, Subsection 2.5 Calibration, Equation 21:


(21) $$\begin{array}{l} \;Y|ℱ(x,S2_{Y})\sim N(ℱ(x,S2_{Y}),\sigma_{Y}^{2}I)\\ Y\sim N(ℳ(x,S2_{Y}),V(x,S2_{Y})+\sigma_{Y}^{2}I) \end{array}$$

## Figure Correction

In the original article, there was a mistake in Figures 8–13 as published. The computer code for the likelihood function for CV(S2) and APD(S2), used for our MCMC simulations, only accounted for the diagonal of the posterior variance matrix $V(x,S2_{Y})$. The corrected Figures 8–13 shown here.

**Figure 8:**
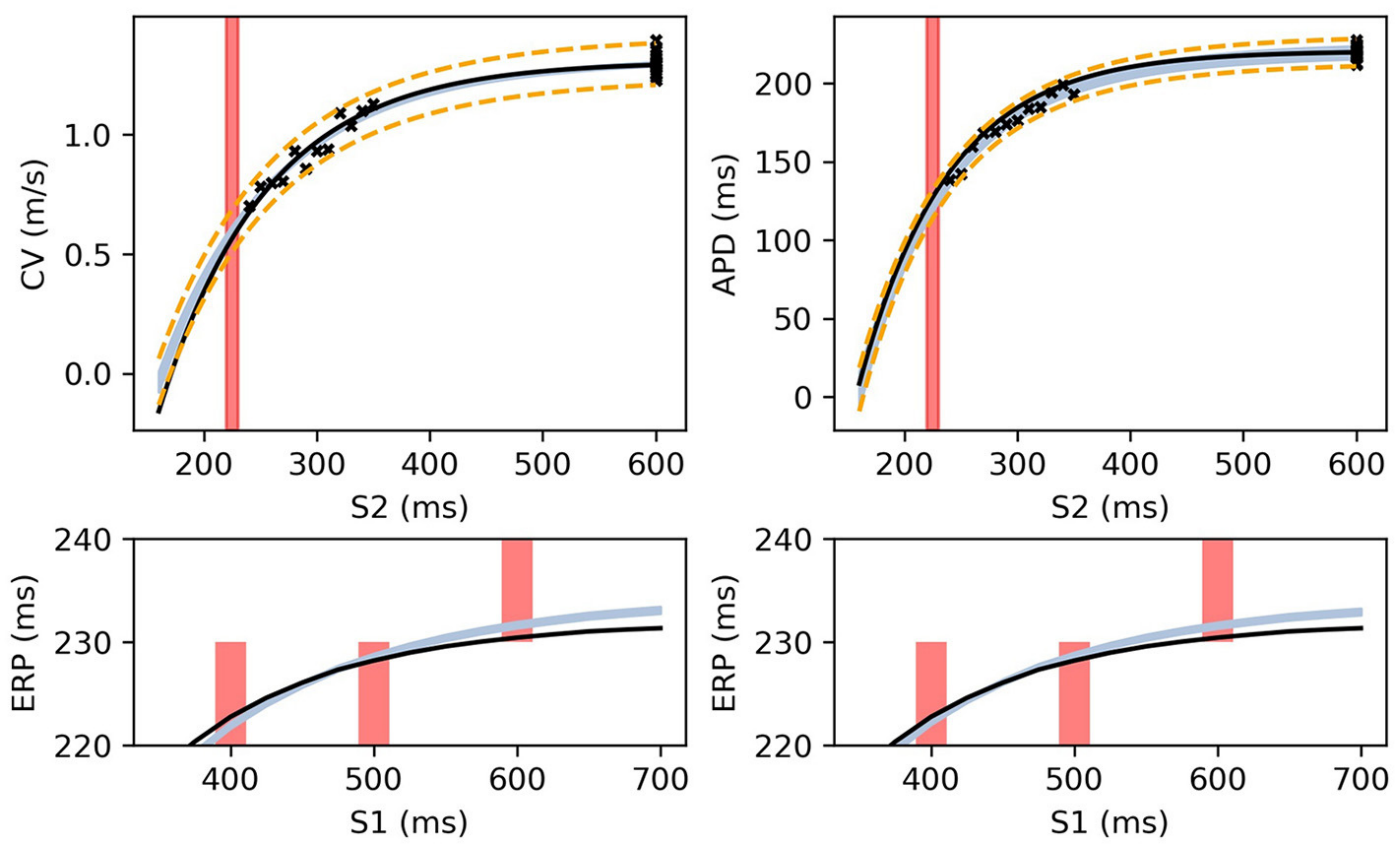
The RCE prediction from maximum a posteriori (MAP) parameter estimates given noisy measurements for (left) CV(S2) and ERP(S1), (right) APD(S2) and ERP(S1), shown as light shaded regions representing RCE 95% confidence intervals. The orange dashed curves show these intervals including the observation error, also learned from MAP fitting. The noisy S2 restitution data are shown as crosses, while the red shaded bars represent observed intervals containing ERP: (top): bars horizontally span ERP(S1:600) interval; (bottom) bars vertically span ERP(S1) interval for several S1. The solid black lines in all plots represent the corresponding ground truth curves.

**Figure 9:**
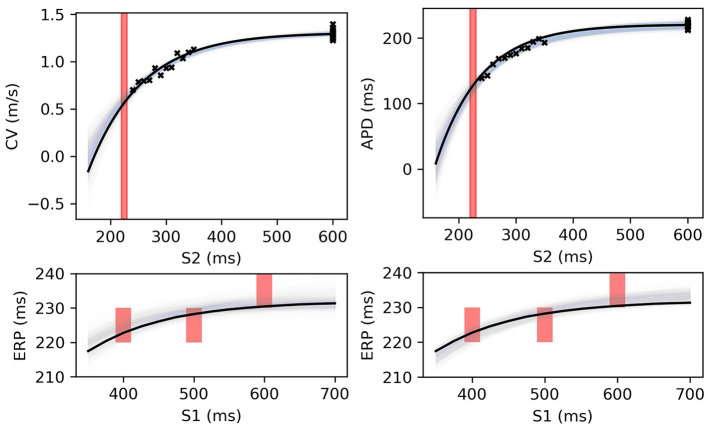
RCE predictions, shown as lightly shaded regions representing 95% confidence intervals, for 100 parameter samples from the posterior distribution given the same measurements shown in Figure 8 [black crosses are noisy S2 restitution data, red bars are observed ERP intervals, (left) MCMC with CV(S2) and ERP(S1) data, (right) MCMC with APD(S2) and ERP(S1) data].

**Figure 10:**
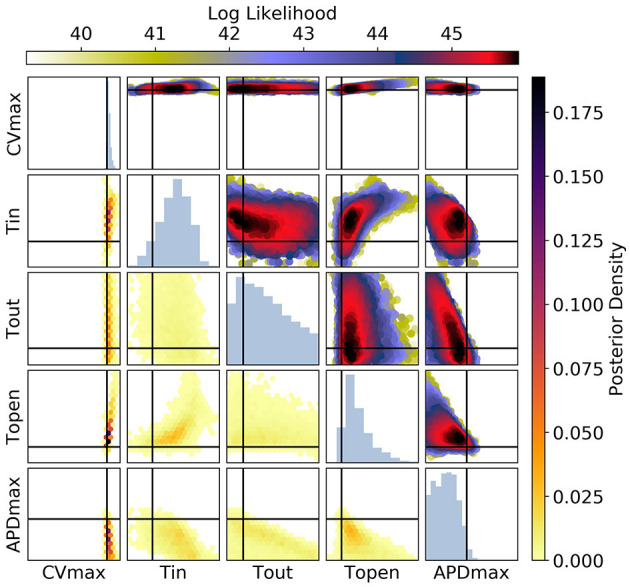
The posterior parameter distribution for fits to CV(S2) and ERP(S1) measurements. The intersection of vertical and horizontal lines mark the true parameter value. The lower diagonal shows the density via hexbin plots, while the upper diagonal shows the log likelihood values for each sample plotted in order of increasing likelihood. The diagonals show the marginal histograms of each parameter.

**Figure 11:**
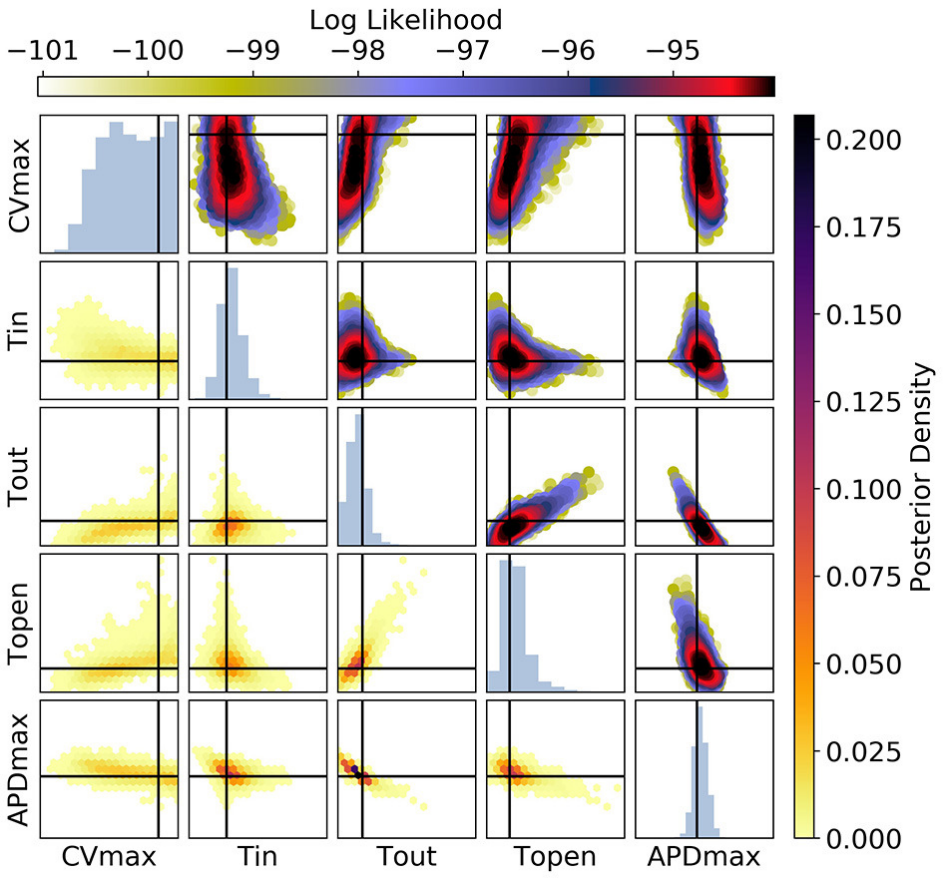
The posterior parameter distribution for fits to APD(S2) and ERP(S1) measurements. The intersection of vertical and horizontal lines mark the true parameter value. The lower diagonal shows the density via hexbin plots, while the upper diagonal shows the log-likelihood values for each sample plotted in order of increasing likelihood. The diagonals show the marginal histograms of each parameter.

**Figure 12:**
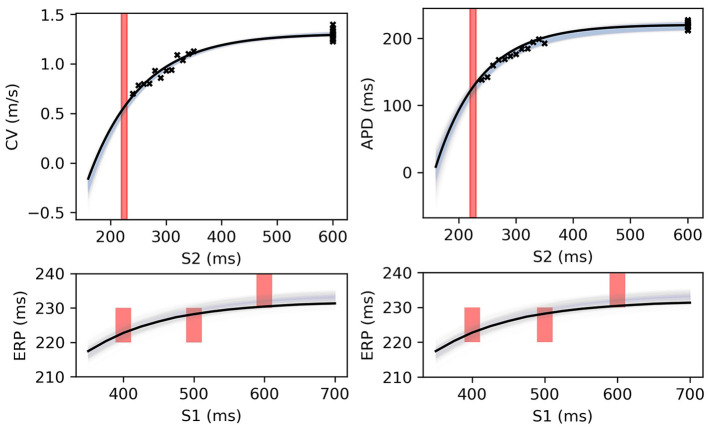
RCE predictions, shown as lightly shaded regions representing 95% confidence intervals, for 100 parameter samples from the posterior distribution given the same measurements shown in Figure 8 (black crosses are noisy S2 restitution data, red bars are observed ERP intervals). MCMC utilized CV(S2), APD(S2), and ERP(S1) data simultaneously, unlike in Figures 8, 9.

**Figure 13:**
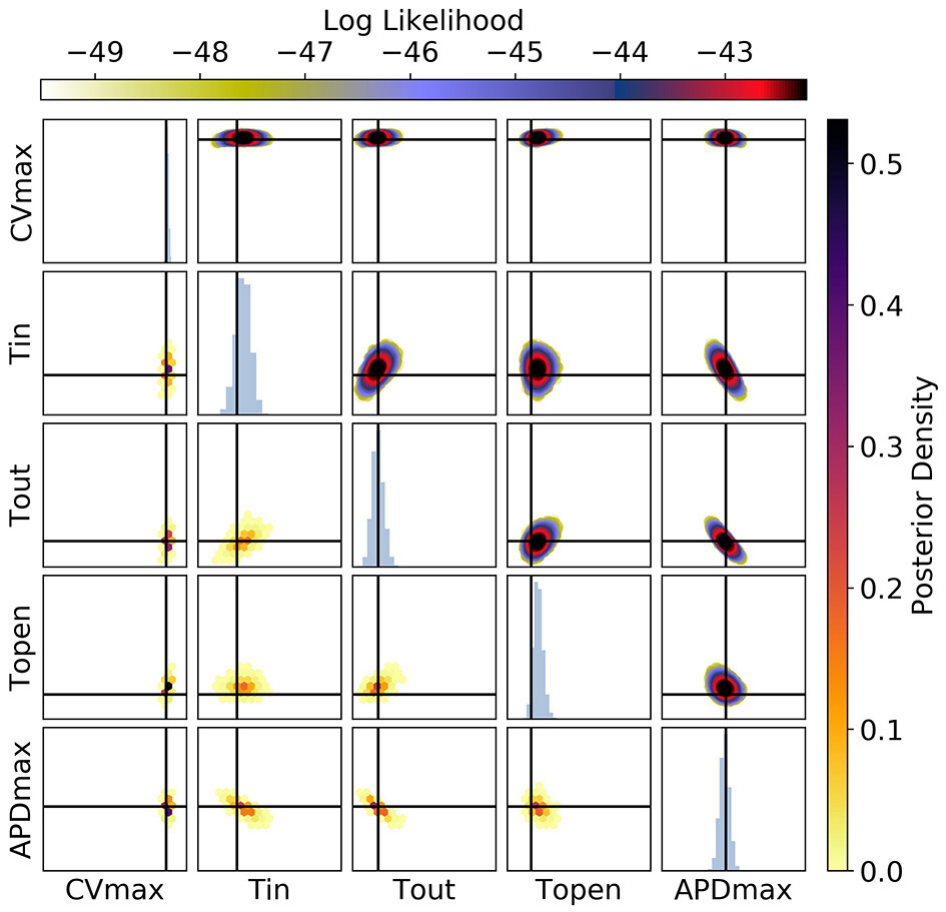
The posterior parameter distribution for calibration to CV(S2), APD(S2), and ERP(S1) measurements simultaneously. The intersection of vertical and horizontal lines mark the true parameter value. The lower diagonal shows the density via hexbin plots, while the upper diagonal shows the log likelihood values for each sample plotted in order of increasing likelihood. The diagonals show the marginal histograms of each parameter.

The authors apologize for this error and state that this does not change the scientific conclusions of the article in any way. The original article has been updated.

## Publisher's Note

All claims expressed in this article are solely those of the authors and do not necessarily represent those of their affiliated organizations, or those of the publisher, the editors and the reviewers. Any product that may be evaluated in this article, or claim that may be made by its manufacturer, is not guaranteed or endorsed by the publisher.

